# Interplay of Sequence, Topology and Termini Charge in Determining the Stability of the Aggregates of GNNQQNY Mutants: A Molecular Dynamics Study

**DOI:** 10.1371/journal.pone.0096660

**Published:** 2014-05-09

**Authors:** Alka Srivastava, Petety V. Balaji

**Affiliations:** Department of Biosciences and Bioengineering, Indian Institute of Technology Bombay, Powai, Mumbai, India; German Cancer Research Center, Germany

## Abstract

This study explores the stabilities of single sheet parallel systems of three sequence variants of ^1^GNNQQNY^7^, N2D, N2S and N6D, with variations in aggregate size (5–8) and termini charge (charged or neutral). The aggregates were simulated at 300 and 330 K. These mutations decrease amyloid formation in the yeast prion protein Sup35. The present study finds that these mutations cause instability even in the peptide context. The protonation status of termini is found to be a key determinant of stabilities; other determinants are sequence, position of mutation and aggregate size. All systems with charged termini are unstable, whereas both stable and unstable systems are found when the termini are neutral. When termini are charged, the largest stable aggregate for the N2S and N6D systems has 3 to 4 peptides whereas N2D mutation supports oligomers of larger size (5-and 6-mers) as well. Mutation at 2^nd^ position (N2S and N2D) results in fewer H-bonds at the mutated as well as neighboring (Gly1/Gln4) positions. However, no such effect is found if mutation is at 6^th^ position (N6D). The effect of Asn→Asp mutation depends on the position and termini charge: it is more destabilizing at the 2^nd^ position than at the 6^th^ in case of neutral termini, however, the opposite is true in case of charged termini. Appearance of twist in stable systems and in smaller aggregates formed in unstable systems suggests that twist is integral to amyloid arrangement. Disorder, dissociation or rearrangement of peptides, disintegration or collapse of aggregates and formation of amorphous aggregates observed in these simulations are likely to occur during the early stages of aggregation also. The smaller aggregates formed due to such events have a variety of arrangements of peptides. This suggests polymorphic nature of oligomers and presence of a heterogeneous mixture of oligomers during early stages of aggregation.

## Introduction

Proteins can self-aggregate into insoluble, fibrillar assemblies called amyloids under conditions that destabilize their native state [1], [2]. These assemblies are associated with a number of neurodegenerative diseases such as Alzheimer's and Huntington's. Despite the diversity in sequence, size, function, and native and secondary structures of amyloid-forming proteins, fibrils formed by them share certain features such as tinctorial properties, morphology, X-ray fiber diffraction pattern and a highly organized internal structure. X-ray fiber diffraction pattern suggests a cross-β spine structure wherein β-strands stack perpendicular to the fiber axis forming β-sheets which grow along the fibril axis [1]–[5]. Formation of amyloid fibrils follows a cooperative, nucleation dependent kinetics with a rate-determining lag phase [1], [6] and has been suggested to be characterized by reversible association of the constituent monomers [7]–[8]. The lag phase ends with the formation of a critical nucleus and can be reduced by seeding with a preformed nucleus [9]. Soluble oligomers that form during the early stages of aggregation are also toxic [10]–[12], besides the mature fibrils that form in later stages [12]–[13].

Amyloid forming property of a protein is thought to lie in specific sequence(s) e.g., ^20^SNNFGAILSS^29^ in islet amyloid polypeptide, ^209^SFNNGDCFILD^219^ in gelsolin, ^7^GNNQQNY^13^ in Sup35 and ^73^GIFQINS^79^ in lysozyme [14]–[16]. Factors that contribute to fibril formation can be extrinsic or intrinsic. Extrinsic factors include concentration, pH, temperature, redox status, ionic strength and co-solutes [17]–[22]. Intrinsic factors are those that depend on the amino acid sequence directly or indirectly. These factors, either alone or in combination with each other, drive the protein/peptide towards amyloid formation. Several experimental and computational studies, especially using peptides, have investigated the correlation between amino acid sequence and aggregation propensity. These have identified sequence-related features such as hydrophobicity, net charge, secondary structure propensities, and pattern of distribution of hydrophobic, charged, aromatic residues as key determinants of aggregation [17], [23]–[24].

The peptide GNNQQNY lies in the N-terminal region of yeast prion protein Sup35 (residues 7 to 13) and is itself capable of forming fibrils independent of the rest of the protein [25]. This peptide forms fibrils as well as a microcrystal, and is the first peptide for which microcrystal structure became available [26]. Based on the microcrystal structure three stages of fibril formation have been envisaged. First, a parallel in-register β-sheet forms where each GNNQQNY peptide is in an extended β strand conformation. Next, two such β-sheets arrange themselves in an anti-parallel orientation. Lastly, many such pairs of sheets associate laterally to form the fibril.

Recent experimental studies on GNNQQNY peptide suggested structural differences between microcrystals and fibrils [27]–[30]. Magic angle spinning NMR studies have found (i) that the peptide assumes different conformations in the fibril and the crystal [30], (ii) significant chemical shift differences between crystals and fibrils [27], and (iii) the coexistence of three distinct conformations of the peptide in fibrillar samples [29]. It has also been found from a combination of experimental techniques (fiber diffraction, electron microscopy (EM), linear dichroism, fluorescence) that the switch from fiber to crystal is accompanied by structural rearrangement; it was suggested that the packing of the GNNQQNY peptides within the initially formed fibrils may differ from the published crystal structures [28]. Taken together, these studies show that, in general, the arrangement of peptides in a fibril as well as the oligomers that form during the lag phase could be different from that in the microcrystal of GNNQQNY.

The inherent insolubility and non-crystalline nature of amyloid fibrils have limited the probing of their atomic structures by techniques such as X-ray crystallography or solution state NMR. Microcrystal structures of only a few amyloidogenic peptides have so far been determined by X-ray crystallography [26], [31]. Consequently, a combination of experimental techniques such as x-ray fiber diffraction, EM, AFM, and solid-state NMR has been used to probe the structures of fibrils. Also, the soluble oligomers formed during the early stages of aggregation are found to be dynamic and short-lived [11], [32] leading to heterogeneity and polymorphism in their structures, topology (arrangement of peptides in the aggregate) and size. This also constrains the use of several techniques for the characterization of early intermediates. Thus, experimental data on their structural features are limited. However, computer simulations can provide data on sizes and time scales that are not accessible to experiments. Hence, atomistic molecular dynamics (MD) simulations have been used extensively to study protein aggregation, to characterize the structures of soluble oligomers and to understand the molecular events leading to their formation [33].

Simulation studies investigate the aggregation mechanism either by monitoring the aggregation behavior of randomly dispersed peptides [34]–[38] and/or the stability and dynamics of pre-formed aggregates [39]–[57]. A variety of peptides have been studied so far such as IAPP, calcitonin, insulin, lysozyme, Sup35, Aβ and β2-microglubulin; amyloid β peptide and its various fragments and sequence variants are the most extensively studied among all these. All the simulations reported so far have considered a homogeneous system and variations are seen in the size and sequence of the peptide in both the approaches. The simulation studies starting from randomly dispersed peptides have fewer variations e.g., the number and conformation of peptides. On the other hand, the studies that start from pre-formed aggregates have more variations e.g., the number of peptides in a sheet, number of sheets, and orientation of strands/sheets, arrangement (planar, in-register, staggered), etc. Despite the differences in the initial structures and the peptide sequence, all these studies indicate that oligomeric aggregate moieties are polymorphic in their size, organization etc., resulting in a heterogeneous pool and hence implying the existence of multiple pathways for aggregation. This is in consonance with recent experimental studies [27]–[30]. The preferred orientation (parallel or anti-parallel), organization (single or double or both), lifetime of aggregate, stabilizing interactions etc. vary with the peptide/system studied.

The availability of high resolution microcrystal structure of GNNQQNY aggregates [26] has triggered a surge of computational studies on this peptide. Many of these simulation studies consider aggregates that have the same structure as found in the microcrystal structure [26] to study the time-dependent evolution and stability of the systems in a crystal-free environment [32]. Recently we have studied the stabilities and dynamics of pre-formed aggregates of this peptide using molecular dynamics simulations with a variety of oligomer structures including single/double sheets and parallel/anti-parallel arrangements along with some other variations such as termini protonation state, twist, temperature [57]. From the study we found that the stabilities of single sheet systems, unlike those of double layer systems, are sensitive to size, protonation status of the peptide termini and the nature of association of peptides viz., parallel or anti-parallel. However, the effect of sequence on the stabilities of these aggregates was not explored. In view of this, in the present study, we have investigated the stabilities and dynamics of single sheet parallel systems, consisting of three sequence variants of ^1^GNNQQNY^7^ using all-atom MD simulation in explicit solvent. The sequence variants chosen are N2D (GDNQQNY), N2S (NSNQQNY) and N6D (GNNQQDY). It has been shown experimentally that Sup35 containing these mutations are defective in amyloid formation and propagation [14]. We are studying the same mutation but in the context of a peptide. Each mutant has been simulated with four aggregate sizes (5 to 8 peptides), in a single arrangement (parallel in-register single β-sheet). The protonation status of the termini is either charged (NH_3_^+^, COO^−^) or neutral (NH_2_, COOH). The systems were simulated at both 300 and 330 K. Thus a total of 50 simulations have been performed in this study (Table 1). The time for each simulation is 50 ns. In addition, six simulations were extended up to 100 ns.

**Table 1:** Simulations performed in this study.

Peptide sequence	GDNQQNY	GSNQQNY	GNNQQDY
Aggregate size i.e., the number of peptides, n, per sheet	5, 6, 7 and 8	5, 6, 7 and 8	5, 6, 7 and 8
Temperature	300 and 330 K	300 and 330 K	300 and 330 K
Simulation name*	nN2D	nN2S	nN6D
Charged termini	5N2D, 6N2D, 7N2D, 8N2D	5N2S, 6N2S, 7N2S, 8N2S	5N6D, 6N6D, 7N6D, 8N6D
Neutral termini¶	5N2D*, 6N2D*, 7N2D*, 8N2D*	5N2S*, 6N2S*, 7N2S*, 8N2S*	5N6D*, 6N6D*, 7N6D*, 8N6D*
Number of simulations†	17	17	16

^*^The following scheme is used for naming the simulations: nXpY, where *n* is the number of peptides per sheet and XpY indicates the nature of mutation. X is the residue in the parent peptide GNNQQNY, p is the position of the mutated residue in the parent peptide and Y is the mutant. The suffix “*” is included to indicate that the N- and C-termini of the peptides are neutral.

¶ Simulations 6N2D*/300 and 7N2S*/330 were performed with two different random numbers as seed.

† Number of simulations  =  (no. of *n* values) × (Temp, 300 or 330 K) × (charged/neutral termini). For GDNQQNY and GSNQQNY peptides, one additional simulation was done.

## Materials and Methods

All the simulations were carried out on quad core Xeon processor systems having 64-bit CentOS 4.3 operating system. GROMACS 4.0.4 was used for MD simulations [58]. VMD [59], Swiss PDB Viewer [60] and PyMol [61] were used for visualization, mutation and rendering of the structures. Graphs were generated using Grace (plasma-gate.weizmann.ac.il/Grace/) and DSSP [62] was used for secondary structure plots. Trajectories were analyzed using GROMACS modules, in-house scripts and MS Excel. Interaction between two tyrosine residues was concluded to be of stacking type if the distance between the ring planes is <0.5 nm. Twist between two peptides was calculated as the angle between the vectors formed by the C-alpha atoms of 2^nd^ and 6^th^ residues of each peptide.

The simulation system has been modeled on the microcrystal structure of GNNQQNY (1YJP.pdb) [26]. The system consists of a single cross-β sheet made up of peptides arranged in parallel to each other. The formation of a single sheet has been proposed to be the first step in fibril organization [26]. In our earlier study on the wild type peptide, the stabilities of single sheet systems varied the most whereas the double layered systems were stable [57]. Also, the protonation status of the termini was found to be an important determinant of stability. In view of these, in the present study, we have simulated single sheet systems with both charged and neutral termini. This also enables studying the effect of termini charge vis-à-vis sequence of the peptide. Many other simulation studies also have considered single sheet systems [44]–[46], [48], [50]–[51], [57]. Termini charges represent the neutral pH condition. Aggregation of peptides with charged termini has been investigated by both simulation [39], [46]–[47] and experimental [28] studies. In the simulation studies, contribution of charged termini has been analyzed by comparing it with neutral termini aggregates. Other factors such as aggregate size (5- to 8-peptide per sheet) and planar in-register arrangement are as in our previous study. Using the same simulation parameters as used for the wild type simulations has facilitated delineating the effect of mutation on stability.

A 7.2 nm cubic box was used with periodic boundary conditions. The minimum distance from the box edge to the peptide was kept to be 1.1 nm to prevent peptides from interacting with their images. The OPLS all-atom force-field was used with LINCS to constrain bonds since all-atom force fields are more accurate than united atom force fields. Particle mesh Ewald algorithm was used to calculate electrostatic interaction with a cut-off of 1.1 nm and a switch function of 1.0 nm. The SHIFT algorithm was used to calculate van der Waals interaction with a cut-off of 1.0 nm and a switch function of 0.9 nm. Berendsen's algorithms were used for temperature and pressure coupling with a coupling constant of 0.1 ps. Peptides and solvent were coupled separately.

The system was energy minimized first in vacuum and again after solvating with single point charge (SPC) water model. Minimization was performed sequentially with steepest descent, conjugate gradient and BFGS algorithms using maximum force as the termination criteria. The force values (in kJ/mol/nm) used for termination for these three methods were 50, 10 and 5 before solvation, and 100, 50 and 10 after solvation respectively. A time step of 2 fs was used to integrate the equations of motion using the leap-frog algorithm. Position restrained MD was performed in NPT ensemble for 2 ns followed by production MD in NVT ensemble for 50 ns. The coordinates of the protein were saved every 250 steps (0.5 ps).

A production MD of 50 ns was deemed adequate since the objective of this study is to monitor stability and dynamics of an already formed aggregate. Moreover, since each simulation is carried out at two temperatures (300 and 330K) the sampling of the conformation space is higher. In fact, simulations with similar objective reported in literature are typically for 10–50 ns [39], [41]–[44], [46]–[48], [50]–[57]. In most of the systems in the present study, changes were indeed observed within this period and hence simulations were not extended further. Some of the systems which are stable at the end of 50 ns were either extended up to 100 ns (six simulations) or restarted with a different random number as the seed for assigning initial velocities (two simulations). Inferences drawn are a collective picture of all the simulations and not based on individual simulations.

## Results

**Figure 1:**
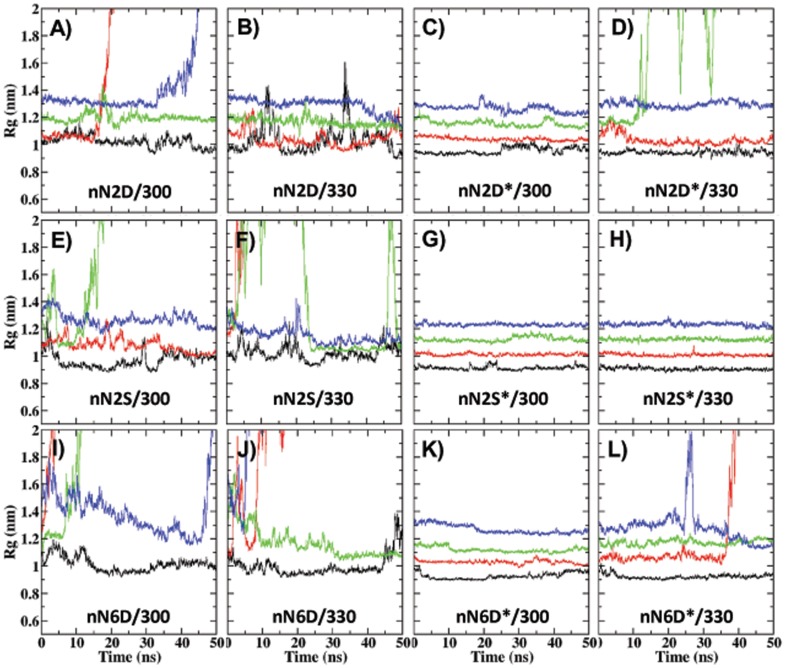
Variations in Rg with time. Name of the simulation is within each graph. Data for aggregates of different sizes are color coded as follows: black, 5 peptides per sheet (n = 5); red, n = 6; green, n = 7; and blue, n = 8. Change in Rg is ≤0.1 nm in all systems with neutral termini except for 7N2D*/330, 6N6D*/330 and 8N6D*/330. In contrast, in systems with charged termini (panels A, B, E, F, I and J), Rg is not constant and three possible types of change (decrease, increase, and large variations) are observed.

In this study, an aggregate is deemed stable if it retains the initial organization of the peptides till the end of 50 ns simulation. Whether or not an aggregate has retained its initial organization is determined first by the magnitude of change in the radius of gyration [ΔR_g_  =  R_g_(final) - R_g_(initial) < 0.1 nm] (Figures 1 and S1). This was followed by visual inspection of the trajectory and analysis of secondary structure and rmsd from the initial structure (Figure S2). It is found that the behavior of the systems is dictated by sequence as well as the protonation status of termini. The parallel arrangement of the peptides in a single sheet leads to arraying of like-charges along the termini leading to repulsion. Thus, all systems with charged termini are unstable, irrespective of the sequence, size of the aggregate and temperature of the simulation. When the termini are neutral, both stable and unstable systems are found and factors such as sequence and size dictate the behavior of the aggregates: most of the aggregates of N2D are unstable whereas aggregates of N2S are stable; aggregates of N6D are stable at lower temperatures. Thus, vis-à-vis aggregate stability, the three mutants differ from the parent peptides and from each other (Table 2).

**Table 2:** Summary table showing the stability of the systems.¶

Charged termini					Neutral Termini			
**Size**	**WT** †	**Mutant N2D**	**Mutant N2S**	**Mutant N6D**	**WT** †	**Mutant N2D**	**Mutant N2S**	**Mutant N6D**
**Simulation temperature: 300 K**								
5	Stable	Unstable Collapse	Unstable Disintegration	Unstable Dissociation	Stable	Unstable Dissociation	Stable	Stable‡
6	Stable	Unstable Dissociation	Unstable Dissociation	Unstable Disintegration	Stable	Stable‡ §	Stable	Stable
7	Unstable Dissociation	Unstable Dissociation	Unstable Dissociation	Unstable Disintegration	Stable	Stable	Stable	Stable
8	Unstable Disintegration	Unstable Dissociation	Unstable Disintegration	Unstable Disintegration	Stable	Unstable Dissociation	Stable‡	Stable
**Simulation temperature: 330 K**								
5	Unstable Dissociation	Unstable Disintegration	Unstable Dissociation	Unstable Dissociation	Stable	Unstable Disorder	Stable‡ ^ζ^	Unstable Rearrangement
6	Unstable Dissociation	Unstable Dissociation	Unstable Disintegration	Unstable Disintegration	Stable	Unstable Dissociation	Stable	Unstable Disintegration
7	Unstable Disintegration	Unstable Disintegration	Unstable Disintegration	Unstable Disintegration	Stable	Unstable Dissociation	Stable‡ §	Stable‡
8	Unstable Disintegration	Unstable Dissociation	Unstable Disintegration	Unstable Disintegration	Stable	Unstable Dissociation	Stable	Unstable Dissociation

† Data for the WT peptide are from Ref.57 and are mentioned here merely to facilitate comparison.

¶ In the case of unstable systems, the key event (disorder, dissociation, disintegration, rearrangement and collapse) is also mentioned.

‡ These simulations were extended up to 100 ns.

§ These simulations were re-initiated with a different random number seed.

ζ Among the extended and re-initiated simulations (total 8), only this system became unstable at ∼95 ns (dissociation).

As mentioned earlier, the stability of an aggregate is based upon the retention of the initial organization till 50 ns. But it is possible that the system may become unstable if the simulations are for a longer duration or re-initiated with a different random number seed. In order to explore this possibility, six of the stable systems were extended up to 100 ns and two stable systems were re-initiated. It is observed that the system is stable in seven out of these eight cases. In the eighth, the system showed instability; one of the edge peptide (A) dissociates at ∼58 ns but moves away from rest of the aggregate at ∼95 ns.

It is observed that the behavior of systems at 300 and 330 K is similar in general although they do differ from each other in details. Events related to instability begin to occur early in the simulation at 330K with few exceptions (8N2S, 5N2D* and 5N2S). For example, in 6N2D system, an edge peptide shows disorder and dissociation. Disorder is observed within 5 ns at 300 K and 2 ns at 330 K. Dissociation is observed after 10 ns at 300 K and within 7 ns at 330 K.

### Unstable systems

**Figure 2:**
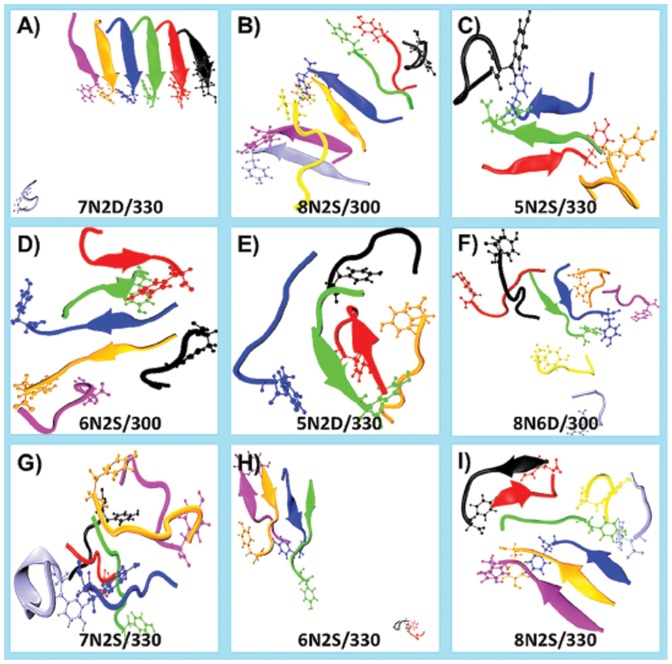
Snapshots showing some of the events observed in unstable systems. Name of the simulation is within each panel. Peptides A, B, C, D, E, F, G, and H are color coded as black, red, green, blue, orange, magenta, ice-blue and yellow, respectively. Panel A (34 ns) shows peptide G (ice-blue) which has dissociated and moved away from the 6-mer. Panel B (7 ns) shows peptide H (yellow) hovering on top of the 7-mer. Panel C (50 ns) shows the smaller aggregate (3-mer) formed by the dissociation of the edge peptides A (black) and E (orange). Also seen is the anti-parallel orientation of peptide B (red) with respect to peptide C (green) as a consequence of re-arrangement event. Panel D (43 ns) shows dissociated edge peptides A (black) and F (magenta). Dissociation has resulted in the formation of a 4-mer (two parallel 2-mers oriented anti-parallel to each other). After dissociation, peptide A has moved from one end (peptide B, red) to the other (peptide E; yellow) whereas peptide F just hovers around peptide E (orange). Panel E (21 ns) shows the amorphous aggregate formed by a gathering of dissociated monomers around a central 2-mer. This is made of peptides B (red) and C (green) that are oriented anti-parallel to each other. Panel F (28 ns) shows an amorphous aggregate formed by the gathering of four dissociated peptides around a central 2-mer. This 2-mer is made of peptides C (green) and D (blue) that are oriented parallel to each other. Panel G (28 ns) shows an amorphous aggregate. The edge peptide G (ice-blue) dissociates (3 ns) and re-associates (26 ns). Panel H (33 ns) shows the 4-mer and 2-mer that are formed by disintegration of the 6-peptide aggregate. The 2-mer has moved away from the 4-mer. In the 4-mer, peptide F (magenta) is anti-parallel to peptide E (orange). Panel I (25 ns) shows the smaller aggregates (3-mer and 2-mer) and monomers formed by the disintegration of the 8-peptide system. The smaller aggregates stick together.

A variety of molecular events such as disorder, dissociation or rearrangement of peptides, and disintegration or collapse of aggregates are observed (Table S1) resulting in the formation of smaller-ordered aggregate (Table S2, Figure 2). They are of varying sizes and their sheet content varies within and across simulations. Simultaneous occurrence of two or more of these events leads to the formation of amorphous aggregates (Figure 2). These may reflect the events during the early stages of aggregation. In systems with neutral termini, the new aggregate is just one or two peptides shorter than the original aggregate (exception 6N6D*/330, 6N2D*/330). However, in systems with charged termini, aggregates of a variety of sizes are sampled (Table S2). The N6D and N2S mutations promote the formation of smaller aggregates (3- and 4-mers), whereas the N2D mutation supports larger aggregates (5- and 6-mers) as well. These smaller ordered oligomers are stable but differ from the microcrystal structure of GNNQQNY in certain respects and the difference is more for the charged termini oligomers (Table S3). The lifetime of the oligomers varies between 5 and 37 ns.

### Stable systems

**Figure 3:**
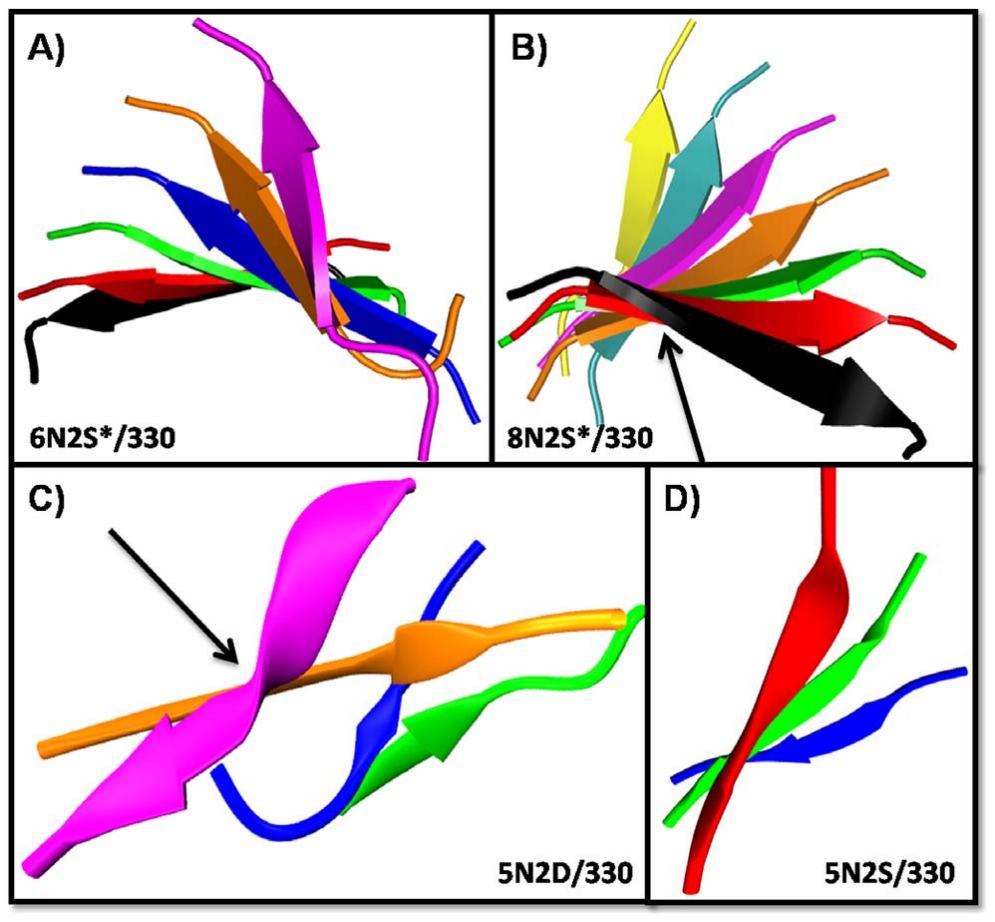
Snapshots showing twist within and across peptides. Name of the simulation is within each panel. Peptides A, B, C, D, E, F, G, and H are color coded as black, red, green, blue, orange, magenta, ice-blue and yellow, respectively. Panels A and B show oligomers in stable systems. Panels C and D show oligomers formed in unstable systems. Twist between peptide pairs is observed in all the simulations. In addition, in some systems, individual peptides are also twisted. The extent of twist varies cf. peptide F (magenta; panel C) with peptide A (black; panel B) (the respective peptides are indicated by arrows).

Characterization of stable systems showed three prominent features: (i) twist within and across peptides (Figures 3, S3, and S4), (ii) temporary disordering/distancing of peptides and (iii) end fraying. However in the microcrystal structure, the peptides are planar (no twist), in an in-register arrangement, with all the residues in sheet conformation (no end fraying) [26]. These differences (Table S4) contribute to the observed rmsd variations (Figure S2). In N2S* systems, all the residues from Ser2 to Tyr7 are in sheet conformation and end fraying is limited to N-terminal Gly (Figures S5a and S5c in File Figure S5). In N2D* systems, end fraying extends to Asp2 and Asn3 also (Figures S5b and S5c in File Figure S5). In contrast to these two systems, in N6D* systems, the C-termini are frayed (minimally for Asp6 and mostly for Tyr7) (Figures S5b and S5c in File Figure S5). End-fraying observed in the N2D* and N6D* systems could be partly due to twisting and partly due to repulsion of Asp residues. Tyr-Tyr stacking is observed in N2S* and N2D* systems due to the absence of C-terminal fraying (Figures S6). The distance between Tyr planes is mostly constant (around 0.5 nm) in N2S* and N2D* systems except that in few systems, stacking is intermittent for the edge peptide pairs. The backbone and side chain H-bonds between pair of peptides are fewer than those in the microcrystal structure of GNNQQNY due to the twisting of peptides and fewer number of residues being in the sheet (Table S4, Figures S7, and S8).

### Mutant vis-à-vis wild type aggregates

The behavior of the aggregates of mutant peptides were compared with those of the wild type peptide simulated under identical conditions in an earlier study [57]. The stabilities of the mutant aggregates vary indicating the effect of sequence on the stability of aggregates. When peptide termini are neutral, the stable mutant aggregates are similar to those of the wild-type if end-frayed residues and edge peptides are excluded; features compared are center of mass distance, twist angle, sheet content, tyrosine stacking, backbone H-bond and side chain H-bond (Table S4, Figure S9).

Most of the events observed in unstable systems of mutants (Figure 2, Table S1) are also seen in the unstable systems of the wild-type peptide [57]. The behavior of unstable wild type systems is similar to that of unstable mutant systems with neutral termini. When the termini are charged, aggregates snap early (<5 ns) and disintegration is rapid in mutant systems.

In a wild-type aggregate and mutant aggregate with neutral termini, peptides remain together as a sheet even after one peptide dissociates. In the case of wild type, dissociation is usually restricted to the C-edge (N- and C-edge are as defined in [57]; briefly, N-edge peptides are those whose N2, Q4 and N6 residues H-bond with the solvent and C-edge peptides are those whose G1, N3, Q5 and Y7 residues H-bond with the solvent). This is true only for a limited number of mutant unstable systems; as in most of the systems, events usually happen very fast and simultaneously. In the wild type systems with charged termini, stability is related to size of the aggregate and stability decreases as size increases from 5 to 8 peptides [57]. In mutant systems stability is both size and sequence dependent. As we have found that with charged termini the N6D and N2S mutations promote the formation of smaller aggregates (max 3-, to 4-mers), whereas the N2D mutation supports larger aggregates as well (5-, to 6-mers). Also the oligomers arising from these events in different simulations vary from one another in their size, orientation, peptide and residues involved in sheet structure.

### Stability vis-à-vis the nature and position of mutation

A mutation can affect the system in many ways depending on the nature and position of mutation. Replacement of Asn by Ser which has a smaller side chain (-CONH_2_ by -OH) has triple effect: Asn forms two backbone and three side chain H-bonds in wild type. After mutation, the number of backbone H-bonds expectedly more or less remains same but side chain H-bonds is mostly reduced to 1. In addition, this mutation reduced the number of H-bonds formed by Gln4 side chain (2–3 in wild type systems, 1–2 in mutant systems) and N-terminal Gly (3 in wild type systems, mostly 1 in mutant system). The number of H-bonds of Tyr side chain also fluctuates between 1 and 0 compared to the almost steady one H-bond in the wild type system (Figures S10a, S10b, S10f and S10g in File Figure S10).

Replacement of polar neutral Asn by the negatively charged Asp (CONH_2_ by COO−) leads to repulsion between the charged side chains. Asn side chain forms 2–3 H-bonds whereas Asp side chain forms zero or at the most one H-bond. In the N2D* systems, there are fewer H-bonds from Gly1, Asp2 and Asn3 backbone, and Asp2 and Gln4 side chains. Similar to N2S mutation, N2D mutation also reduces the number of H-bonds formed by Gly1 backbone (3 in WT; 1 or 2 in mutant) and Gln4 side chain (2–3 in WT and 1–2 in mutant). In addition, Asn2 forms 2–3 side-chain H-bonds in wild type systems, whereas Asp2 forms zero or just 1 side chain H-bond (Figures S10c, S10f, and S10g in File Figure S10). There is no such effect if the Asn→Asp mutation is at the 6th position: the total number of backbone or side-chain H-bonds is highest in this mutant system (Figures S10d, S10e, S10f and S10g in File Figure S10).

The two Asp mutants showed contrasting stabilities: N2D mutant aggregates are more stable when termini are charged whereas N6D mutant aggregates are more stable if the termini are neutral (Table 2, Table S2). With charged termini, proximity of the terminal positive and side chain negative charges stabilize the aggregate in N2D systems. In N6D systems, there are two negative charges in tandem leading to increased repulsion which explains the larger destabilization and formation of smaller oligomers. When the termini are neutral, there is an array of side chain negative charges. This, together with loss of backbone H-bonds (due to end fraying) and lack of side chain interactions (terminal residue is Gly), destabilizes the N2D* aggregates. In N6D*, stability arises due to Tyr7 and Gln5 side chains interaction and absence of N-terminal end fraying.

## Discussion

In the present study, stabilities of oligomeric aggregates in relation to their sequence, size and the protonation status of peptide termini have been investigated by MD simulations. The pre-formed aggregates consist of a single sheet of peptides arranged in parallel to each other. Three sequence variants of GNNQQNY have been considered: N2D, N6D and N2S. In Sup35, these mutations are known to decrease the aggregation propensity [14]. Herein the same mutations are studied but in the context of a peptide. MD simulations show that these mutations cause instability in peptides also.

### Sequence - aggregation propensity relationship: a paradox?

Amyloid forming proteins do not share sequence or native structural similarity. In fact, amyloid formation appears to be an intrinsic property of a protein/peptide. Under appropriate conditions, any protein/peptide can form amyloid [1]–[4]. This seems to suggest that amyloid formation depends on conditions rather than sequence. On the other hand, several studies have shown that even a single mutation can dramatically alter aggregation propensity [52], [53], [60]–[69] showing that amyloid formation is not totally sequence independent. Thus, sequence and aggregation propensity seem to have a paradoxical relationship but this can be resolved if one takes into consideration factors such as the context of the peptide (exists independently or is part of a protein) and context/nature of the mutation (position/property) along with extrinsic factors (pH etc.). It can be argued that for every mutation that leads to amyloid formation, there exists a condition under which it is no longer amyloidogenic and vice versa.

In the present study, it is evident that “condition” overrides “sequence” in determining aggregation propensities. For example, aggregates of all sizes of the N2S mutant are stable in neutral termini condition implying that this is not a destabilizing mutation. However, this same mutation is destabilizing in charged termini condition (Figures 1 and S1, Table 2). It is known that substitution by a charged residue can prevent fibril formation [64], [67]–[69]. Hence, mutating a polar neutral to a charged residue is expected to be destabilizing. This indeed is observed in the present study since aggregates of N2S mutants are more stable than those of N2D and N6D mutants in the neutral termini condition. However when peptide termini are charged, it is observed that, for the N2D mutant, oligomers of larger size (5-mers and 6-mers) are stable whereas for the N2S mutant, only smaller size aggregates (2-, 3- and 4-mers; Table S2) are stable. This once again shows that “condition” can override the “sequence” effect.

Between the two Asp mutants N2D and N6D, aggregates of the former are more stable in the charged termini condition whereas aggregates of the latter are more stable if the termini are neutral (Table 2, Table S2). In fact, even wild type systems in single sheet parallel arrangement showed stability in neutral termini condition and instability in a size dependent manner in charged termini condition [57]. Thus, protonation status of the termini (charged or neutral) is an important determinant of the stabilities of the aggregates in this arrangement. When the protonation status of the termini is not the determining factor, sequence of the peptide becomes the primary determinant of stability.

When the termini are neutral, aggregates of the polar neutral mutant (N2S*) are more stable than those of the charged mutant (N2D* and N6D*) (Table2). When the mutant residue is identical, the location of the mutation becomes important in determining the stability. The effect of Asn→Asp mutation depends on the position and termini charge. This mutation is more destabilizing at the 2^nd^ position than at the 6^th^ in case of neutral termini as judged by the number of unstable systems in each mutation (Figures 1 and S1, Table 2). However, the opposite is true in case of charged termini as evident from the characteristics of the newly formed oligomer (size, sheet content and lifetime) in the unstable systems (Table S3). Positional dependence of mutation in proteins and amyloid peptides has been reported earlier also: it has been found from MD simulations that the location where the residues Pro-Gly are inserted is an important determinant of the conformational dynamics of polyQ [66]. A combined positional scanning mutagenesis, MS, CD and EM study on the peptide STVIIE found that the effect of different types of mutations on amyloidogenicity depends on the location of the mutation within the amyloidogenic peptide; for example, charged residues are allowed only at the termini [67].

The key role played by the protonation status of termini in determining the topology and stability of oligomers was observed for the wild type systems also [57]. Another simulation study on GNNQQNY also reached a similar conclusion since it was found that when termini are charged, anti-parallel arrangement is preferred over parallel and vice versa [39]. It has been found that the protonation status of termini does not affect the stabilities of double layered systems by us [57] as well as others [46], [47]. This suggests that termini charge is an important factor in the early stages (i.e., when peptides are forming single sheets) in comparison to later stages (when double sheets are formed). Alternatively, charged termini may force peptides into a double layered arrangement early on. Fiber diffraction, EM, LD and fluorescence studies on the fibrillation of GNNQQNY with charged termini found similarities as well as differences between the structures of fibers and crystals [28]; since the topology of the peptides in the aggregates is not known, further assessment of whether this effect is related to the way peptides are arranged in the aggregate cannot be made. However, taken together, these observations suggest that the residues flanking the amyloidogenic sequence in a protein may play a critical role in fibril formation.

### Sequence - termini charge - topology relationship

Parallel arrangement of peptides offers some advantages while having some limitations. It leads to a continuous array of identical groups and depending on the type of group, such an arrangement can either favor a stable aggregate or be detrimental. For example, the presence of a polar neutral group such as –CONH_2_ (from the side chain of Asn/Gln; forms a polar zipper) or an aromatic group such as phenyl (from the side chain of Phe/Tyr; forms stacking interaction) can stabilize the aggregate [70]–[72]. On the other hand, presence of an array of charged groups such as –COO^−^/NH_3_^+^ (from the side chain of Asp/Glu/Arg/Lys) will be detrimental to stability. As found in studies on *Aβ peptide* that the charged status of *Asp/Lys residues* determines *the conformation of the Aβ peptide aggregate and that “unsatisfied” charge is incompatible with fibril-like conformers* [64], [67], [69]. Consequently, in the present simulations, various types of re-arrangements (e.g. twisting, staggering, out-of-register alignment or parallel to anti-parallel switching) or formation of smaller size aggregates (by dissociation or disintegration) are observed (Table S1, Table S2). Although many amyloid structures have been proposed to consist of parallel peptides [26], [73]–[74], this arrangement imposes a constraint on the sequence of the peptide. Charged termini, which prove to be detrimental in parallel arrangement, can be advantageous if the orientation of peptide changes from parallel to anti-parallel. This is observed many times in the present study when parallel to anti-parallel transition occurs either for a peptide pair or for a pair of dimers (Figure 2). Even for the wild-type peptide, aggregates with anti-parallel arrangement are more stable than those with parallel peptides when termini are charged [57]. From a MD simulation study on the same peptide GNNQQNY, it has been reported that parallel and anti-parallel orientations are equally distributed for the smaller aggregates when the termini are charged [39]. However, it is possible that the distribution changes when one considers larger aggregates. Overall, it can be concluded that the topology of the aggregate is intricately related to sequence and termini charge: a peptide may not be able to form stable aggregates of certain topologies, but changing the topology (parallel to anti-parallel or vice versa) and/or invoking additional stabilizing factors (single to double layer) may promote aggregation.

In the microcrystal structure of GNNQQNY, a steric-zipper is formed by inter-digitations of the Asn and Gln side chains (N2, Q4 and N6) in the dry interface between two sheets. In view of this, most of the mutation studies on this peptide have targeted these residues. Mutating these residues to Gly or Ala resulted in unstable aggregates [46], [47] implying that the steric zipper is essential for the stability of double layered systems. However, such mutations caused instability of even single sheet systems [47]. This can be interpreted to mean that the polar zipper is required for the stability of single sheet parallel systems whereas the steric zipper is important to double layered systems for this peptide.

### Possible molecular events during the early stages of aggregation

The instability of aggregates is caused by one or more factors such as termini charge, sequence and size. The instability results in the formation of smaller aggregates with different arrangements of peptides such as parallel, anti-parallel, crossed, or double layered arrangements. Only a few of these smaller oligomers resemble the aggregate in the microcrystal structure of GNNQQNY. The smaller (2- to 6-mers) oligomers resemble the microcrystal structure only in certain respects e.g., inter-peptide distance and sheet conformation. Collectively, these observations suggest the presence of a heterogeneous mixture of oligomers in the early stages of aggregation. An oligomer of given size displays polymorphism in the arrangement of peptides, number of residues in sheet and end-fraying. Such heterogeneity [75]–[77] and polymorphism [76]–[79] have been proposed by other simulation studies as well. These are in tune with the results of recent experimental studies on GNNQQNY peptide which found an increased complexity in the behavior of the fibrillar form of GNNQQNY [27]–[30].

There are many simulation studies on the stability and dynamics of pre-formed aggregates which vary in size, orientation and organization. These studies also differ from each other in the force field used e.g., united atom force field such as GROMOS96 43A1 [39]–[41], [46]–[47], [50]–[51], [55], CHARMM 19 [80], and all atom force field such as CHARMM PARAM22 [42]–[43], [48], OPLS [57], Amber ff94 [45], Amberparam99 [56], Amber99SB [52]–[54] and simulation methods such as constant temperature MD [41]–[47], replica exchange MD [39]–[40], [55], [80], and reaction path annealing [62]. Despite these differences, these studies broadly converge in their conclusions: factors such as termini charge, size of the aggregate and simulation temperature affect aggregates with single sheet much more than those which are double layered. It has also been concluded that the double layer is more stable than the single sheet and twisting of strands is integral to the aggregates. The inference that the broad conclusions are not sensitive to simulation protocol is reinforced by the recent study in which pre-formed aggregates of tau peptide and of insulin peptide were simulated using different force fields (Gromos96 43A1, Amber99SB and CHARMM27) [81].

In conclusion, the present all atom MD simulation study on single sheet parallel aggregates of GNNQQNY sequence variants reveals the importance of charged termini, sequence, and location of residues for the stability of such aggregates. The protonation status of termini is a major determining factor of the stability when peptides are arranged in parallel in a single sheet in the aggregate. A single point mutation in this peptide not only alters the interaction at the site of mutation but also at neighboring positions. Asn side chain is more suited than Ser side chain to stabilize aggregates through the formation of a polar zipper. This is because of the difference in the lengths of the two side chains and the presence of separate donor (NH2) and acceptor (C = O) groups in Asn. Unsatisfied H-bond potential of a charged residue imparts instability to the aggregate. Thus, a charged residue must be located in the peptide in such a way that it can interact favorably with its neighbors. In the present simulations, various types of re-arrangements (e.g. twisting, staggering, out-of-register alignment or parallel to anti-parallel switching) or formation of smaller size aggregates (by dissociation or disintegration) are observed. This strengthens the view that amyloid fibril formation goes through a polymorphic heterogeneous phase in the early stages of aggregation.

## Supporting Information

Figure S1 Variations in Rg with time in the extended simulations (top and middle panel) and re-initiated simulations (bottom panel). Names of the simulations are within each graph. Change in Rg is ≤ 0.1 nm in all these systems except for 5N2S*/330, where one of the edge peptide (A) dissociates at ∼58 ns, hover on top of the rest of the aggregate and ultimately move away at ∼95 ns.
(PDF)

Figure S2 **a** Variations in RMSD with time in stable systems with neutral termini. Name of the simulation is within each panel. All N2D* systems are unstable at 330 K. Data for aggregates of different sizes are color coded as follows: black, 5 peptides per sheet (n = 5); red, n = 6; green, n = 7; and blue, n = 8. In the simulation 5N6D*/330, peptide E becomes anti-parallel within 5 ns but the system remains stable after that. Hence, data for this simulation are included along with those for stable systems in this and the subsequent supplementary figures. **b** Variations in RMSD in the extended simulations (top and middle panel) and re-initiated simulations (bottom panel). Name of the simulation is within each panel. Large change in rmsd for 5N2S*/330 is due to dissociation of an edge peptide. In rest of the systems changes in rmsd is related to the appearance of twist within and between peptides.
(PDF)

Figure S3 **a** Variations in twist angle with time in stable systems. Name of the simulation is within each panel. Twist angle between different peptide pairs are color coded as follows: black, between A and B, red =  B and C, green  =  C and D, blue  =  D and E, orange  =  E and F, sea green  =  F and G and magenta  =  G and H. **b** Variations in twist angle with time in the extended simulations (top and middle panel) and re-initiated simulations (bottom panel). Name of the simulation is within each panel.
(PDF)

Figure S4 **a** Average twist between neighboring peptides in stable systems. Name of the simulation is within each panel. All N2D* systems are unstable at 330 K. In the simulation 5N6D*/330 (panel D, black), peptide E becomes anti-parallel to peptide D. The twist between these two peptides is (135°) is out of the range of this graph. **b** Average twist between neighboring peptides in the extended simulations (top and middle panel) and re-initiated simulations (bottom panel). Name of the simulation is within each panel.
(PDF)

Figure S5 **a** Average Cα-Cα distance between equivalent residues of neighboring peptides in N2S*systems. Name of the simulation is within each panel. **b** Average Cα-Cα distance between identical residues of neighboring peptides in N2D* and N6D* stable mutant systems. Name of the simulation is within each graph. **c** Average Cα-Cα distance between equivalent residues of neighboring peptides in in the extended simulations (top and middle panel) and re-initiated simulations (bottom panel). Name of the simulation is within each panel.
(PDF)

Figure S6 **a** Variations in the distance between planes of Tyr rings of neighboring peptides in stable systems. Name of the simulation is within each panel. The distance between two Tyr planes of different peptide pairs are color coded as follows: black, between A and B, red =  B and C, green  =  C and D, blue  =  D and E, orange  =  E and F, sea green  =  F and G and magenta  =  G and H. **b** Variations in the distance between planes of Tyr rings of neighboring peptides in in the extended simulations (top and middle panel) and re-initiated simulations (bottom panel). Name of the simulation is within each panel.
(PDF)

Figure S7 **a** Total number of H-bonds in stable systems. Panels A to E, backbone-backbone H-bonds and panels F to J, side chain-side chain. Name of the simulation is within each panel. Data for aggregates of different sizes are color coded as follows: black, 5 peptides per sheet (n = 5); red, n = 6 green, n = 7, blue, n = 8. **b** Total number of backbone-backbone H-bonds in the extended simulations (top and middle panel) and re-initiated simulations (bottom panel). Name of the simulation is within each panel. **c** Total number of side chain-side chain H-bonds in the extended simulations (top and middle panel) and re-initiated simulations (bottom panel). Name of the simulation is within each panel.
(PDF)

Figure S8 **a** Average number of backbone-backbone H-bonds between pairs of peptides in stable systems. Name of the simulation is within each panel. **b** Average number of side chain-side chain H-bonds between pair of peptides in stable systems with neutral termini. Name of the simulation is within each panel. **c** Average number of backbone-backbone H-bonds between pairs of peptides in the extended simulations (top and middle panel) and re-initiated simulations (bottom panel). Name of the simulation is within each panel. **d** Average number of side chain-side chain H-bonds between pairs of peptides in between pairs of peptides in the extended simulations (top and middle panel) and re-initiated simulations (bottom panel). Name of the simulation is within each panel.
(PDF)

Figure S9 Comparison of the total number of backbone and side chain H-bonds in mutant aggregates with those in WT aggregates (data from ref 57). Data averaged over all the peptides and entire trajectory. Data compared for only the stable systems.
(PDF)

Figure S10 **a** Average number of backbone-backbone H-bonds formed by different residues of the peptide in N2S* systems. Name of the simulation is within each panel. Data for edge peptides, which have only one partner, have not been shown. **b** Average number of side chain-side chain H-bonds formed by different residues of the peptide in N2S* systems. Name of the simulation is within each panel. Data for edge peptides, which have only one partner, have not been shown. **c** Average number of backbone-backbone and side chain-side chain H-bonds formed by different residues of the peptide in stable N2D* systems. Name of the simulation is within each panel. Data for edge peptides, which have only one partner to interact with, have not been shown. **d** Average number of backbone-backbone H-bonds formed by different residues of the peptide in the stable N6D* systems. Name of the simulation is within each panel. Data for edge peptides, which have only one partner to interact with, have not been shown. **e** Average number of side chain-side chain H-bonds formed by different residues of the peptide in the stable N6D* systems. Name of the simulation is within each panel. Data for edge peptides, which have only one partner to interact with, have not been shown. **f** Average number of backbone-backbone H-bonds formed by different residues of the peptide in between pairs of peptides in the extended simulations (top and middle panel) and re-initiated simulations (bottom panel). Name of the simulation is within each panel. Data for edge peptides, which have only one partner, have not been shown. **g** Average number of side chain-side chain H-bonds formed by different residues of the peptide in in between pairs of peptides in the extended simulations (top and middle panel) and re-initiated simulations (bottom panel). Name of the simulation is within each panel. Data for edge peptides, which have only one partner, have not been shown.
(PDF)

Table S1 Summary of events observed in unstable systems.
(PDF)

Table S2 Sizes of the smaller ordered aggregates that form in unstable systems.
(PDF)

Table S3 Comparison of the structural characteristics of smaller aggregates formed by unstable systems of mutant peptides with the microcrystal structure of GNNQQNY.
(PDF)

Table S4 Comparison of the stable systems with the Wild Type systems and microcrystal structure of GNNQQNY.
(PDF)
