# Supplementary material for: Interplay of Sequence, Topology and Termini Charge in Determining the Stability of the Aggregates of GNNQQNY Mutants: A Molecular Dynamics Study
Source: PLoS One. 2014 May 9;9(5):e96660. doi: 10.1371/journal.pone.0096660 (PMC4015988; doi:10.1371/journal.pone.0096660)
Supplement: Figure S1 — Variations in Rg with time in the extended simulations (top and middle panel) and re-initiated simulations (bottom panel). Names of the simulations are within each graph. Change in Rg is ≤ 0.1 nm in all these systems except for 5N2S*/330, where one of the edge peptide (A) dissociates at ∼58 ns, hover on top of the rest of the aggregate and ultimately move away at ∼95 ns. (PDF) [file pone.0096660.s001.pdf]

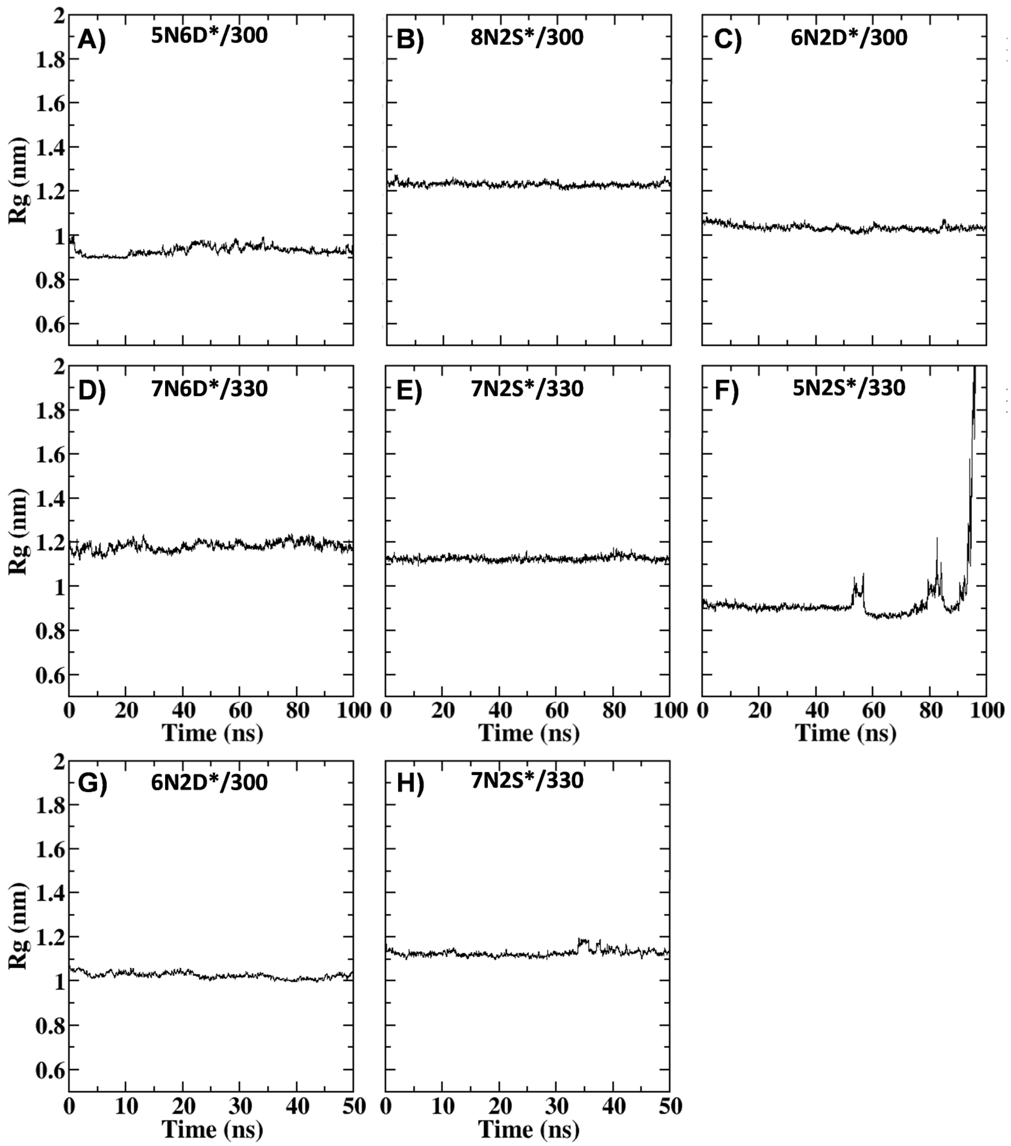

Figure S1 Variations in Rg with time in the extended simulations (top and middle panel) and re-initiated simulations (bottom panel). Names of the simulations are within each graph. Change in Rg is ≤ 0.1 nm in all these systems except for 5N2S*/330, where one of the edge peptide (A) dissociates at ~58 ns, hover on top of the rest of the aggregate and ultimately move away at ~95 ns.
