# Supplementary material for: Interplay of Sequence, Topology and Termini Charge in Determining the Stability of the Aggregates of GNNQQNY Mutants: A Molecular Dynamics Study
Source: PLoS One. 2014 May 9;9(5):e96660. doi: 10.1371/journal.pone.0096660 (PMC4015988; doi:10.1371/journal.pone.0096660)
Supplement: Figure S2 — a Variations in RMSD with time in stable systems with neutral termini. Name of the simulation is within each panel. All N2D* systems are unstable at 330 K. Data for aggregates of different sizes are color coded as follows: black, 5 peptides per sheet (n = 5); red, n = 6; green, n = 7; and blue, n = 8. In the simulation 5N6D*/330, peptide E becomes anti-parallel within 5 ns but the system remains stable after that. Hence, data for this simulation are included along with those for stable systems in this and the subsequent supplementary figures. b Variations in RMSD in the extended simulations (top and middle panel) and re-initiated simulations (bottom panel). Name of the simulation is within each panel. Large change in rmsd for 5N2S*/330 is due to dissociation of an edge peptide. In rest of the systems changes in rmsd is related to the appearance of twist within and between peptides. (PDF) [file pone.0096660.s002.pdf]

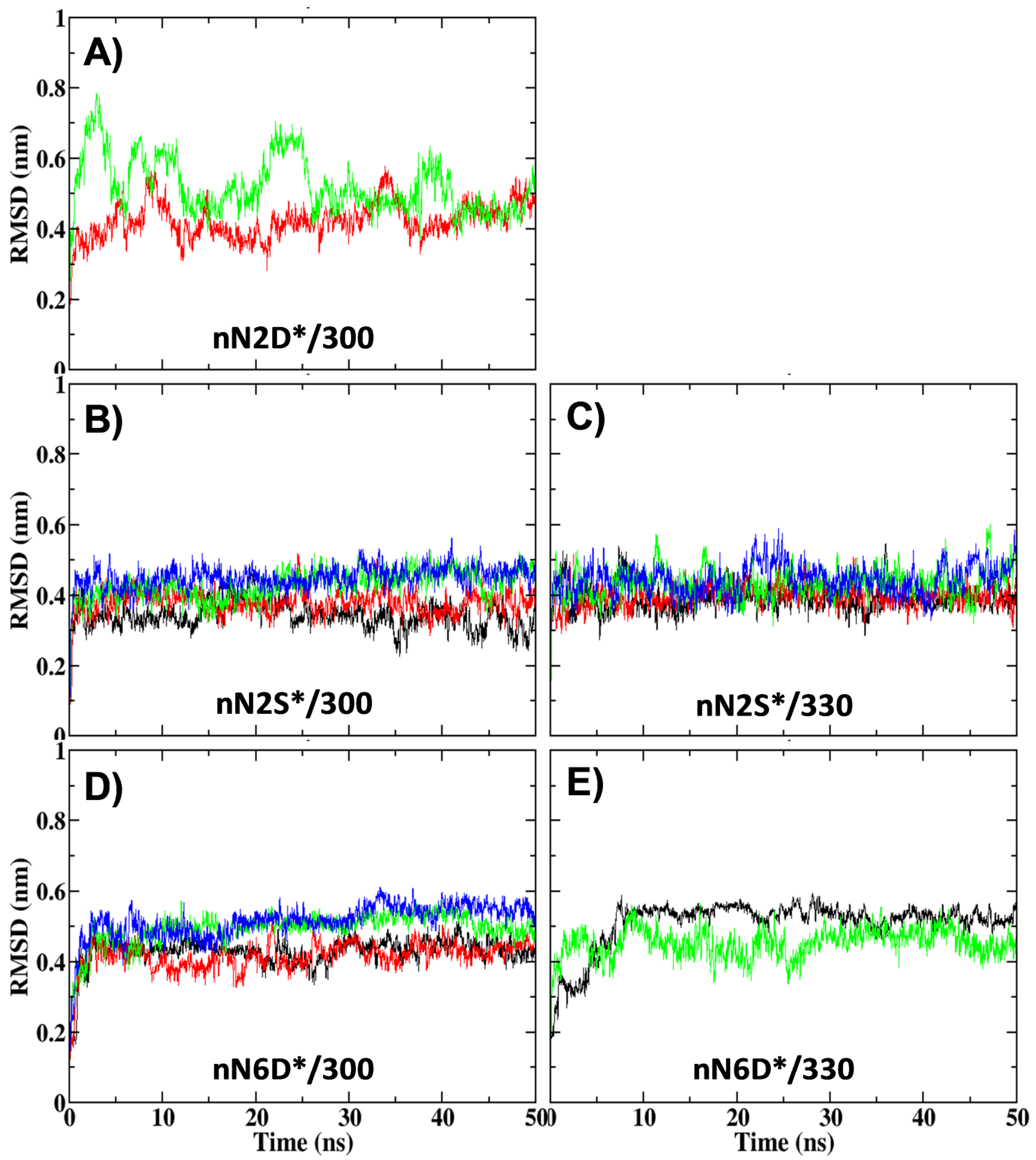

Figure S2a Variations in RMSD with time in stable systems with neutral termini. Name of the simulation is within each panel. All N2D* systems are unstable at 330 K. Data for aggregates of different sizes are color coded as follows: black, 5 peptides per sheet (n=5); red, n=6; green, n=7; and blue, n=8. In the simulation 5N6D*/330, peptide E becomes anti-parallel within 5 ns but the system remains stable after that. Hence, data for this simulation are included along with those for stable systems in this and the subsequent supplementary figures.

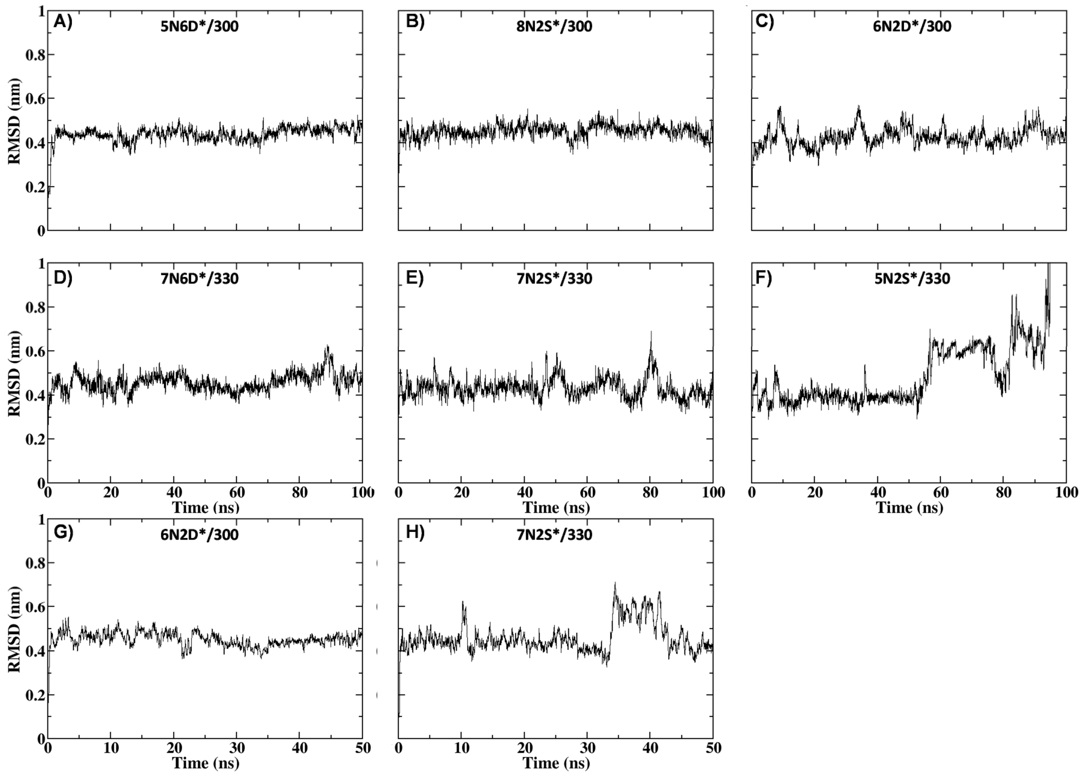

Figure S2b Variations in RMSD in the extended simulations (top and middle panel) and re-initiated simulations (bottom panel). Name of the simulation is within each panel. Large change in rmsd for 5N2S*/330 is due to dissociation of an edge peptide. In rest of the systems changes in rmsd is related to the appearance of twist within and between peptides.
