# Supplementary material for: Interplay of Sequence, Topology and Termini Charge in Determining the Stability of the Aggregates of GNNQQNY Mutants: A Molecular Dynamics Study
Source: PLoS One. 2014 May 9;9(5):e96660. doi: 10.1371/journal.pone.0096660 (PMC4015988; doi:10.1371/journal.pone.0096660)
Supplement: Figure S3 — a Variations in twist angle with time in stable systems. Name of the simulation is within each panel. Twist angle between different peptide pairs are color coded as follows: black, between A and B, red = B and C, green = C and D, blue = D and E, orange = E and F, sea green = F and G and magenta = G and H. b Variations in twist angle with time in the extended simulations (top and middle panel) and re-initiated simulations (bottom panel). Name of the simulation is within each panel. (PDF) [file pone.0096660.s003.pdf]

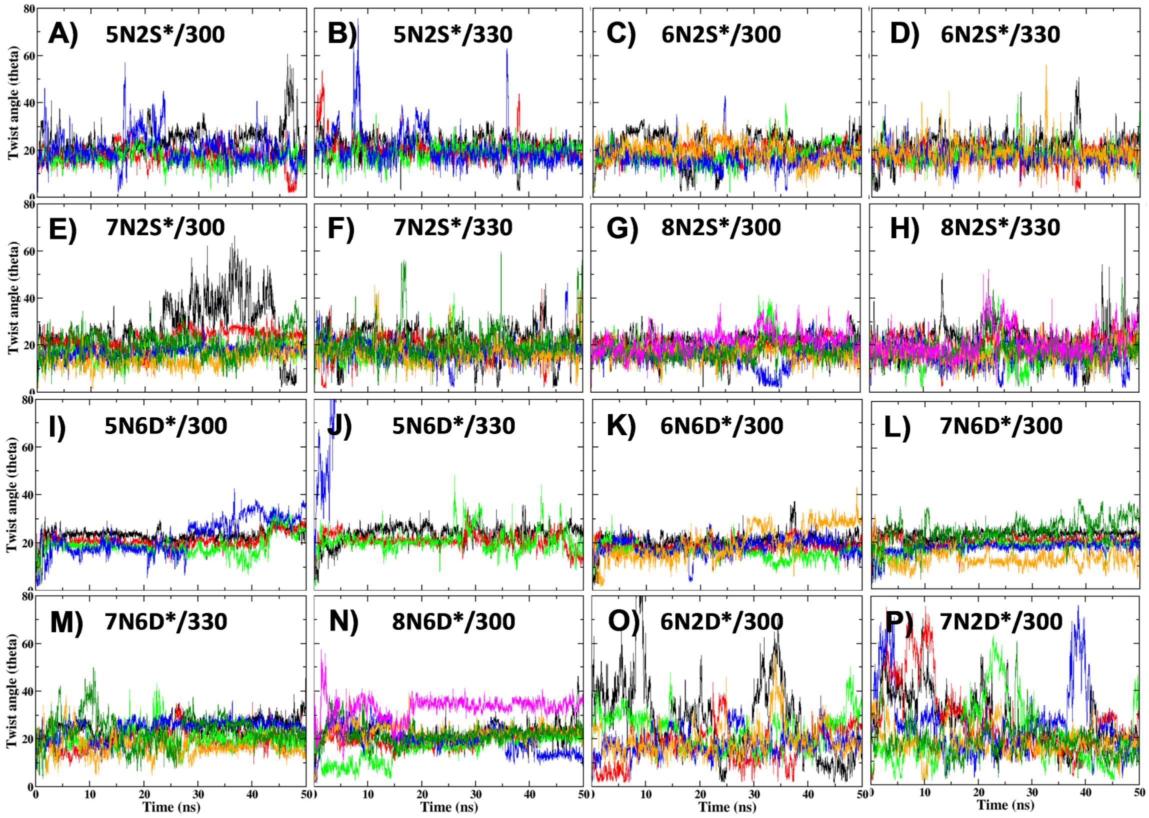


**Figure S3a** Variations in twist angle with time in stable systems. Name of the simulation is within each panel. Twist angle between different peptide pairs are color coded as follows: black, between A and B, red= B and C, green = C and D, blue = D and E, orange = E and F, sea green = F and G and magenta = G and H.

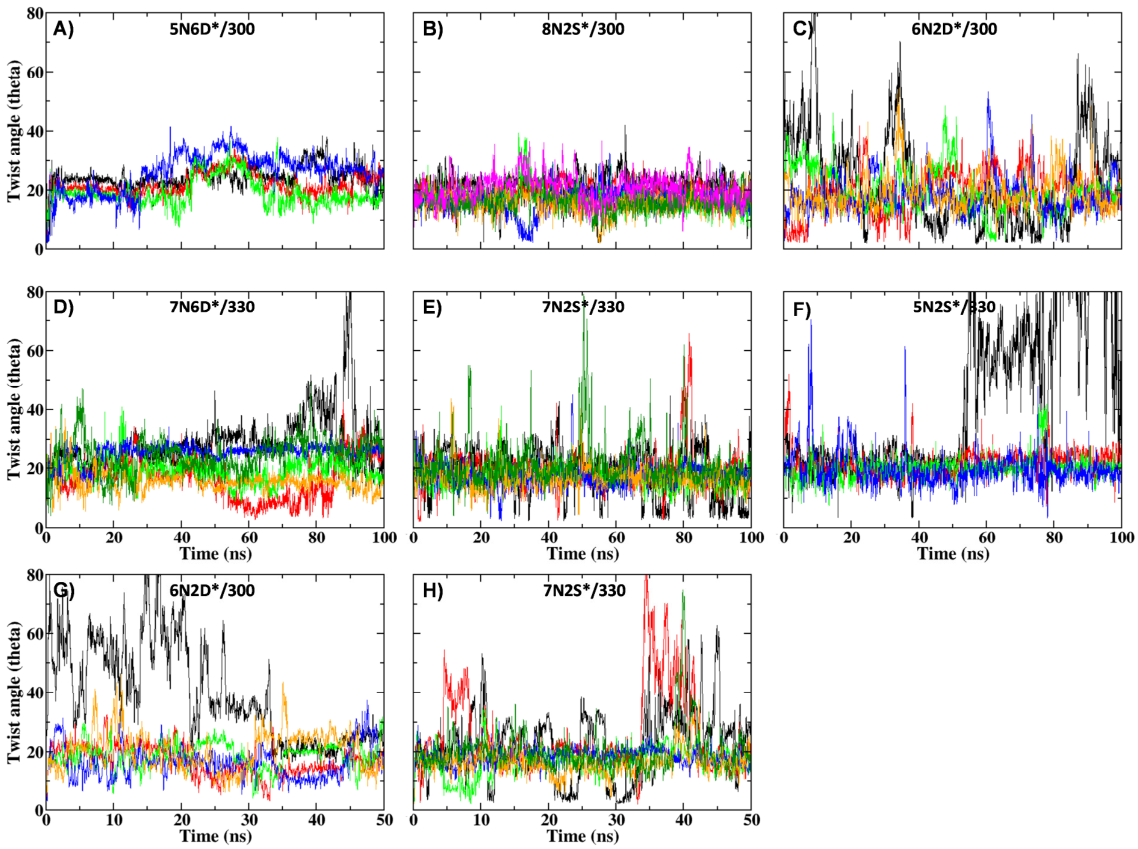


**Figure S3b** Variations in twist angle with time in the extended simulations (top and middle panel) and re-initiated simulations (bottom panel). Name of the simulation is within each panel.
