# Supplementary material for: Interplay of Sequence, Topology and Termini Charge in Determining the Stability of the Aggregates of GNNQQNY Mutants: A Molecular Dynamics Study
Source: PLoS One. 2014 May 9;9(5):e96660. doi: 10.1371/journal.pone.0096660 (PMC4015988; doi:10.1371/journal.pone.0096660)
Supplement: Figure S4 — a Average twist between neighboring peptides in stable systems. Name of the simulation is within each panel. All N2D* systems are unstable at 330 K. In the simulation 5N6D*/330 (panel D, black), peptide E becomes anti-parallel to peptide D. The twist between these two peptides is (135°) is out of the range of this graph. b Average twist between neighboring peptides in the extended simulations (top and middle panel) and re-initiated simulations (bottom panel). Name of the simulation is within each panel. (PDF) [file pone.0096660.s004.pdf]

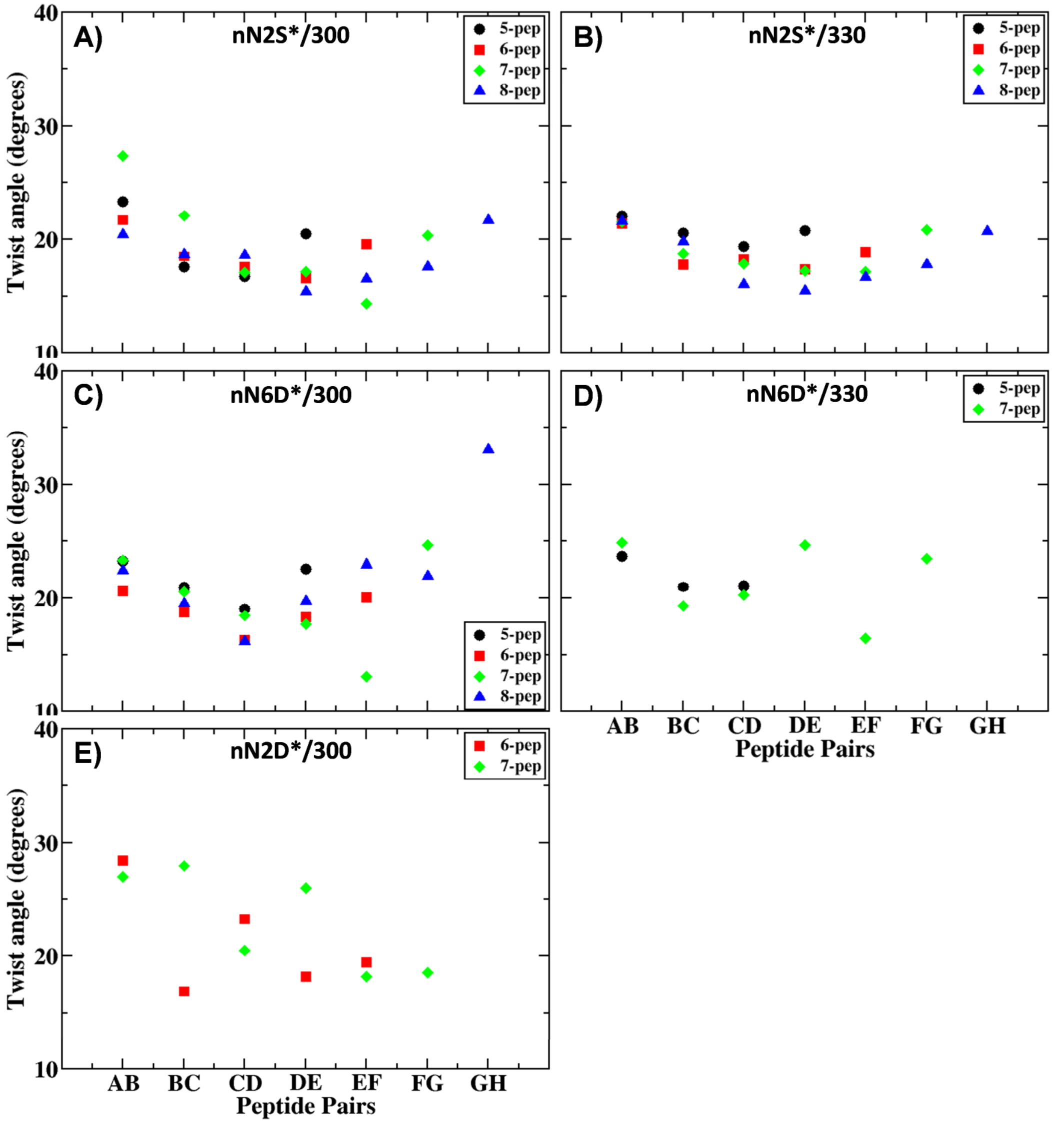

Figure S4a Average twist between neighbouring peptides in stable systems. Name of the simulation is within each panel. All N2D* systems are unstable at 330 K. In the simulation 5N6D*/330 (panel D, black), peptide E becomes anti-parallel to peptide D. The twist between these two peptides is (135°) is out of the range of this graph.

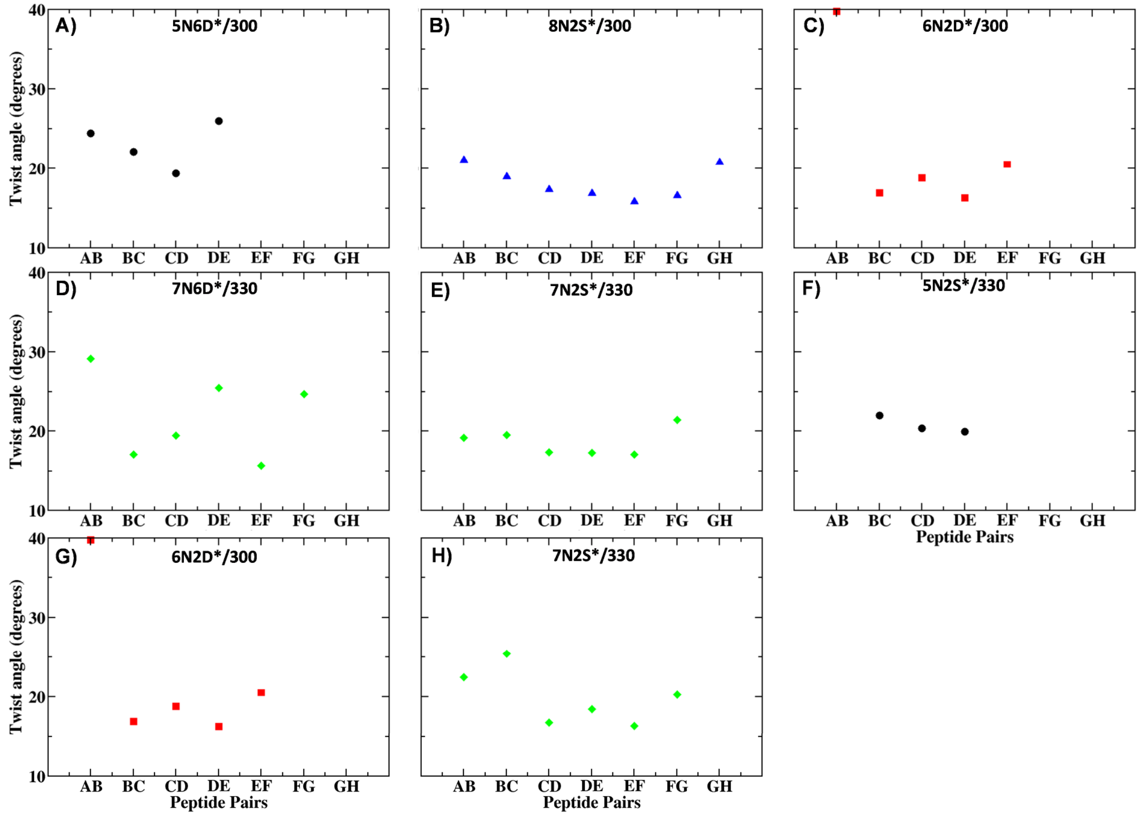

Figure S4b Average twist between neighbouring peptides in the extended simulations (top and middle panel) and re-initiated simulations (bottom panel). Name of the simulation is within each panel.
