# Supplementary material for: Interplay of Sequence, Topology and Termini Charge in Determining the Stability of the Aggregates of GNNQQNY Mutants: A Molecular Dynamics Study
Source: PLoS One. 2014 May 9;9(5):e96660. doi: 10.1371/journal.pone.0096660 (PMC4015988; doi:10.1371/journal.pone.0096660)
Supplement: Figure S5 — a Average Cα-Cα distance between equivalent residues of neighboring peptides in N2S*systems. Name of the simulation is within each panel. b Average Cα-Cα distance between identical residues of neighboring peptides in N2D* and N6D* stable mutant systems. Name of the simulation is within each graph. c Average Cα-Cα distance between equivalent residues of neighboring peptides in in the extended simulations (top and middle panel) and re-initiated simulations (bottom panel). Name of the simulation is within each panel. (PDF) [file pone.0096660.s005.pdf]

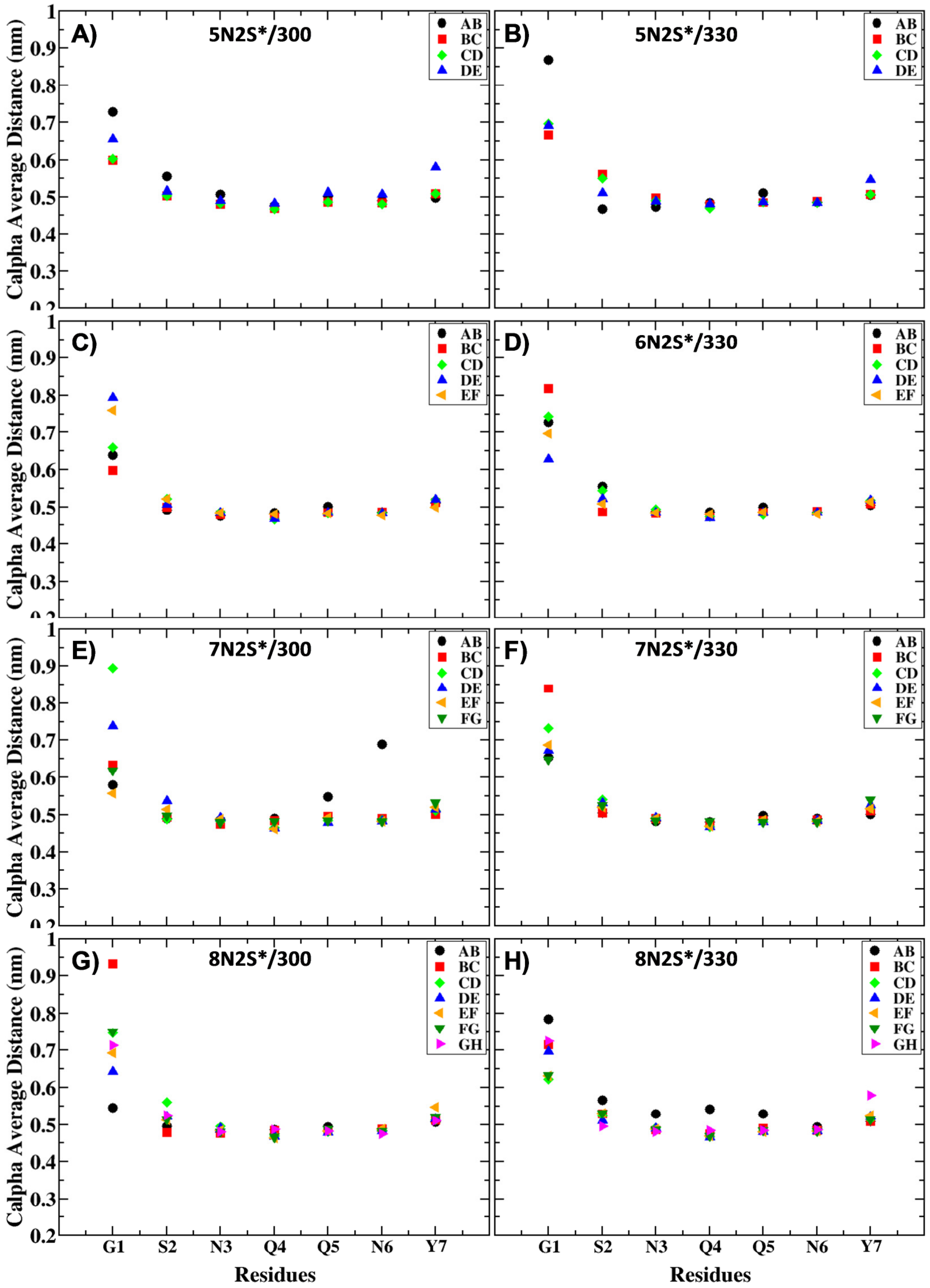

Figure S5a Average Cα-Cα distance between equivalent residues of neighbouring peptides in N2S*systems. Name of the simulation is within each panel.

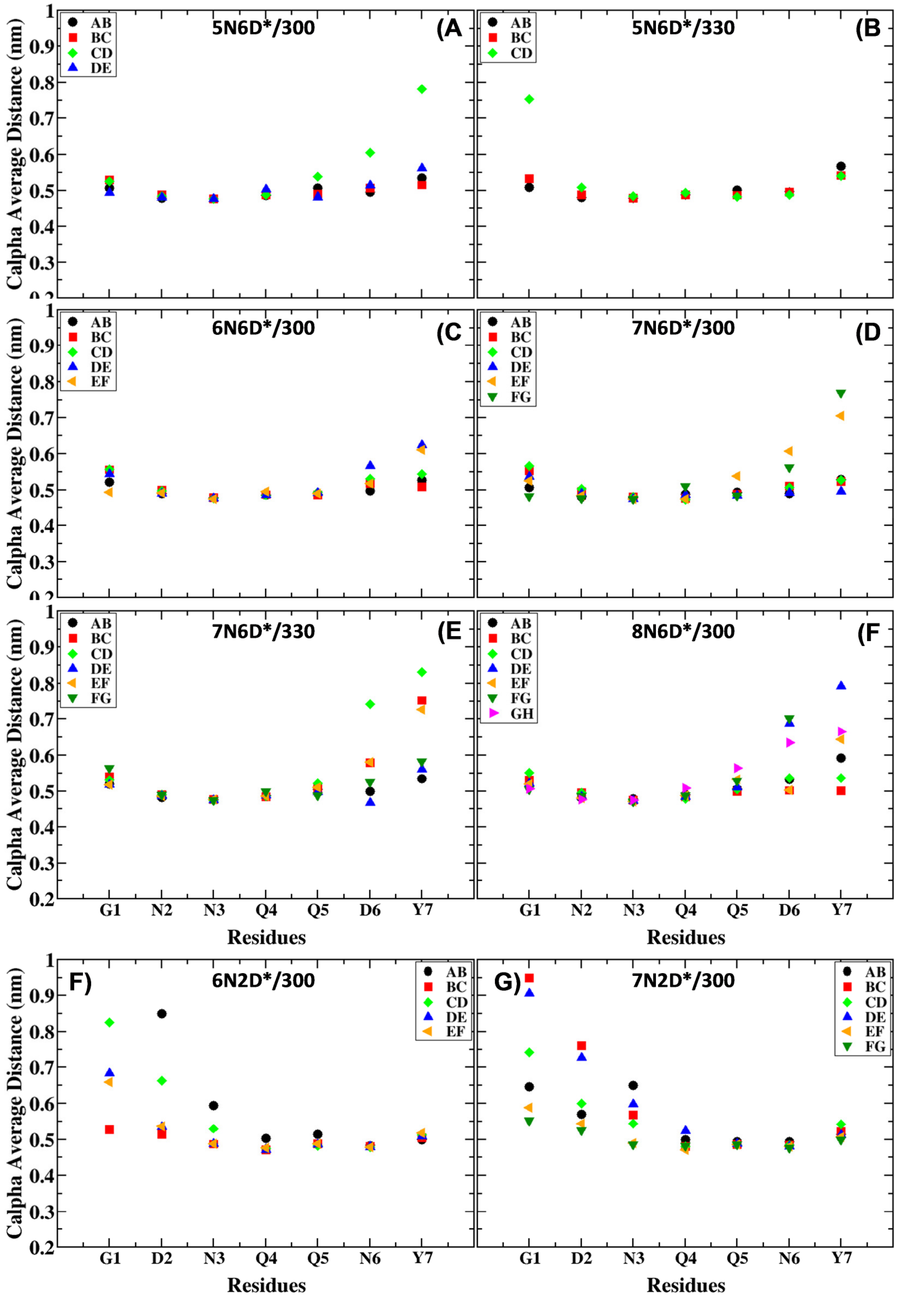

Figure S5b Average Cα-Cα distance between identical residues of neighbouring peptides in N2D* and N6D* stable mutant systems. Name of the simulation is within each graph.

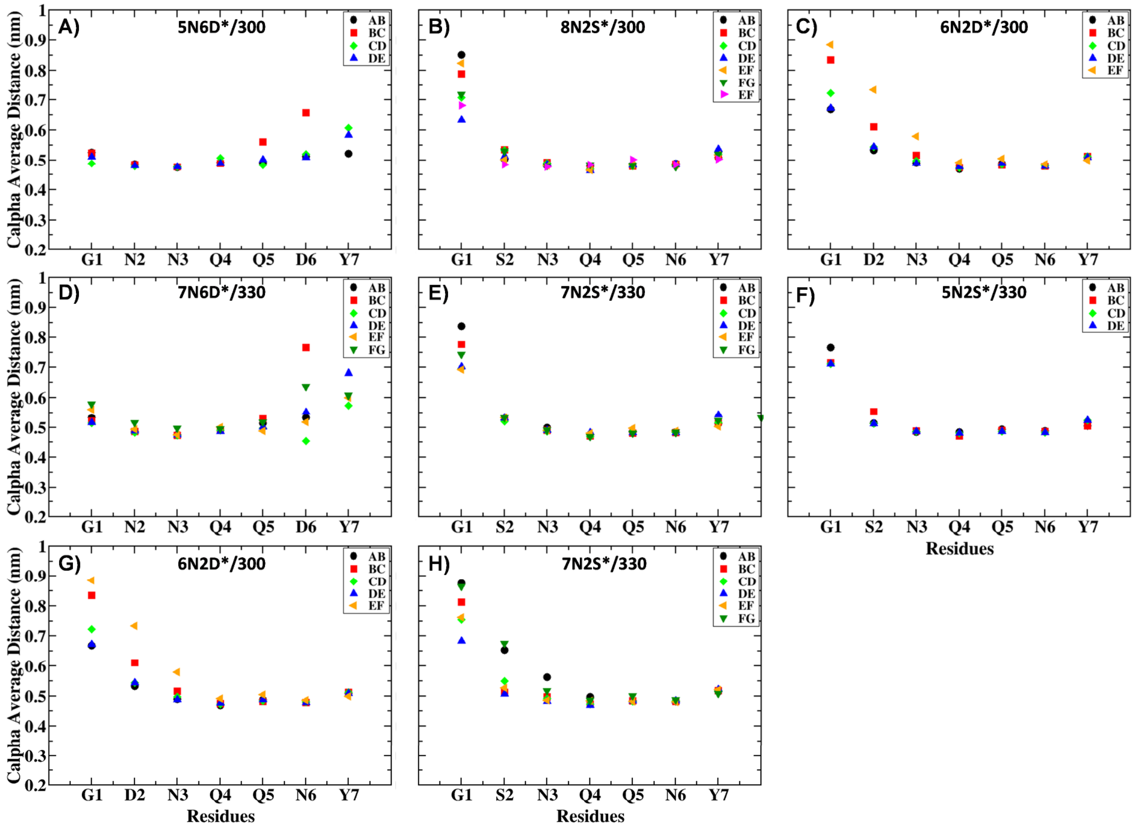

Figure S5c Average Cα-Cα distance between equivalent residues of neighbouring peptides in in the extended simulations (top and middle panel) and re-initiated simulations (bottom panel). Name of the simulation is within each panel.
