# Supplementary material for: Interplay of Sequence, Topology and Termini Charge in Determining the Stability of the Aggregates of GNNQQNY Mutants: A Molecular Dynamics Study
Source: PLoS One. 2014 May 9;9(5):e96660. doi: 10.1371/journal.pone.0096660 (PMC4015988; doi:10.1371/journal.pone.0096660)
Supplement: Figure S6 — a Variations in the distance between planes of Tyr rings of neighboring peptides in stable systems. Name of the simulation is within each panel. The distance between two Tyr planes of different peptide pairs are color coded as follows: black, between A and B, red = B and C, green = C and D, blue = D and E, orange = E and F, sea green = F and G and magenta = G and H. b Variations in the distance between planes of Tyr rings of neighboring peptides in in the extended simulations (top and middle panel) and re-initiated simulations (bottom panel). Name of the simulation is within each panel. (PDF) [file pone.0096660.s006.pdf]

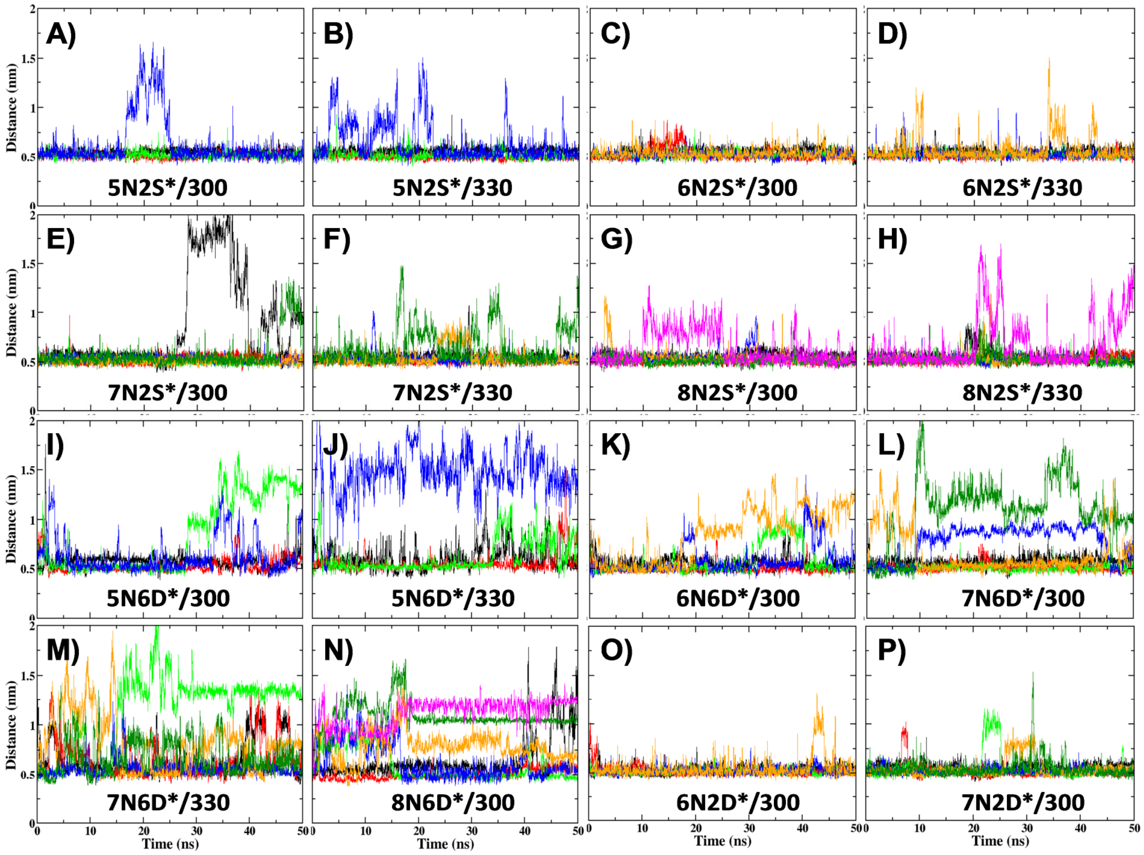

Figure S6a Variations in the distance between planes of Tyr rings of neighbouring peptides in stable systems. Name of the simulation is within each panel. The distance between two Tyr planes of different peptide pairs are color coded as follows: black, between A and B, red= B and C, green = C and D, blue = D and E, orange = E and F, sea green = F and G and magenta = G and H.

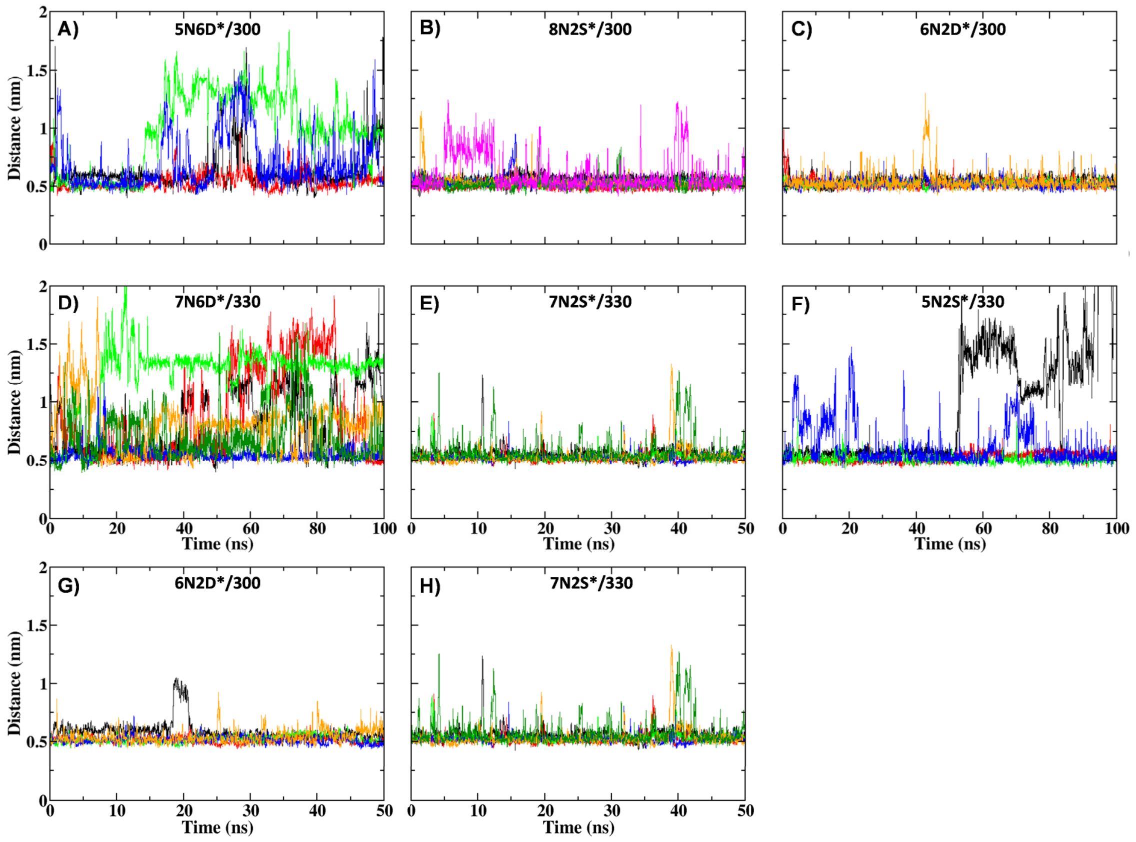

Figure S6b Variations in the distance between planes of Tyr rings of neighbouring peptides in in the extended simulations (top and middle panel) and re-initiated simulations (bottom panel). Name of the simulation is within each panel.
