# Supplementary material for: Interplay of Sequence, Topology and Termini Charge in Determining the Stability of the Aggregates of GNNQQNY Mutants: A Molecular Dynamics Study
Source: PLoS One. 2014 May 9;9(5):e96660. doi: 10.1371/journal.pone.0096660 (PMC4015988; doi:10.1371/journal.pone.0096660)
Supplement: Figure S7 — a Total number of H-bonds in stable systems. Panels A to E, backbone-backbone H-bonds and panels F to J, side chain-side chain. Name of the simulation is within each panel. Data for aggregates of different sizes are color coded as follows: black, 5 peptides per sheet (n = 5); red, n = 6 green, n = 7, blue, n = 8. b Total number of backbone-backbone H-bonds in the extended simulations (top and middle panel) and re-initiated simulations (bottom panel). Name of the simulation is within each panel. c Total number of side chain-side chain H-bonds in the extended simulations (top and middle panel) and re-initiated simulations (bottom panel). Name of the simulation is within each panel. (PDF) [file pone.0096660.s007.pdf]

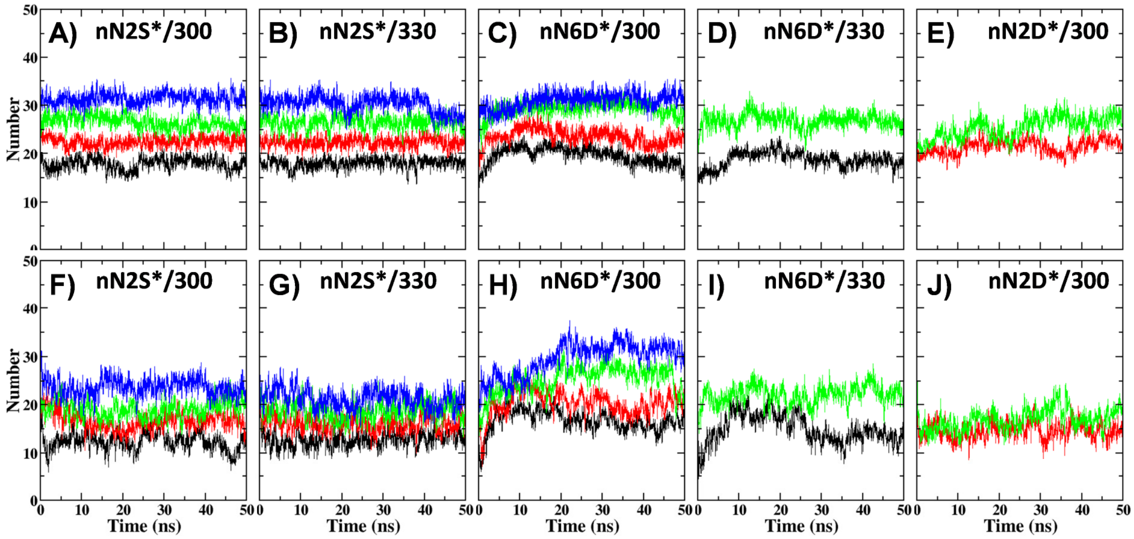

Figure S7a Total number of H-bonds in stable systems. Panels A to E, backbone-backbone H-bonds and panels F to J, side chain-side chain. Name of the simulation is within each panel. Data for aggregates of different sizes are colour coded as follows: black, 5 peptides per sheet (n=5); red, n=6 green, n=7, blue, n=8.

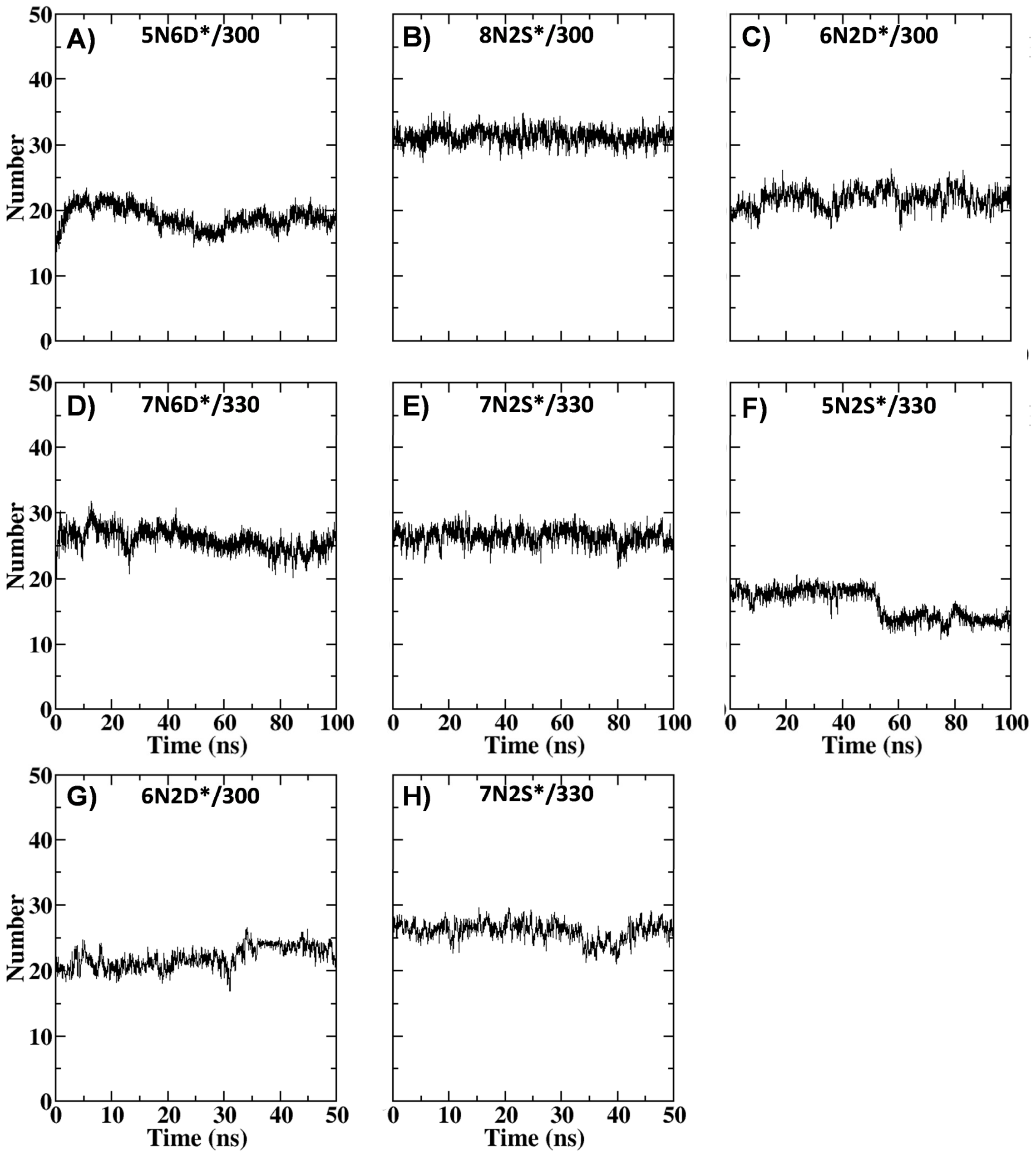

Figure S7b Total number of backbone H-bonds in the extended simulations (top and middle panel) and re-initiated simulations (bottom panel). Name of the simulation is within each panel.

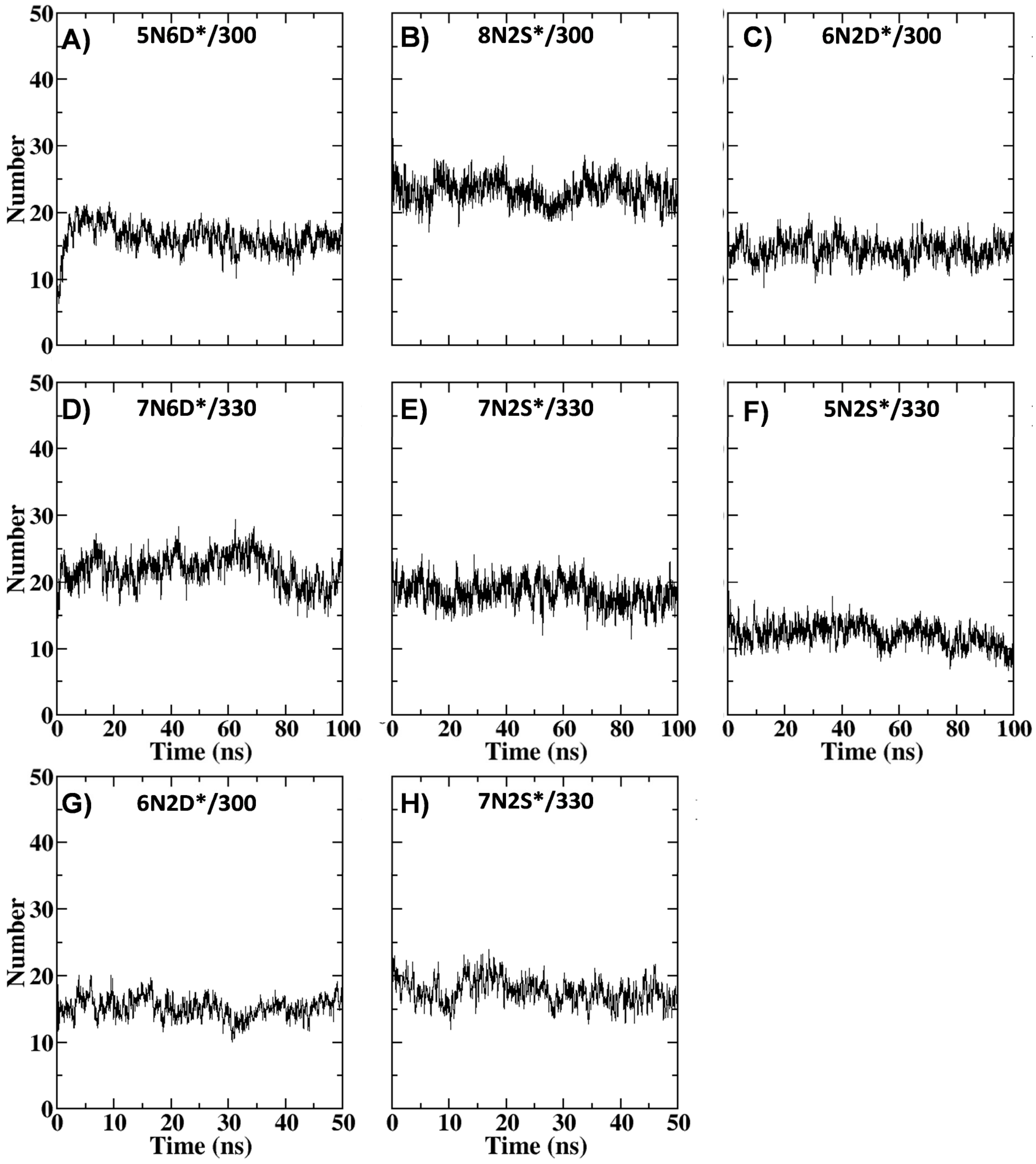

Figure S7c Total number of side chain H-bonds in the extended simulations (top and middle panel) and re-initiated simulations (bottom panel). Name of the simulation is within each panel.
