# Supplementary material for: Interplay of Sequence, Topology and Termini Charge in Determining the Stability of the Aggregates of GNNQQNY Mutants: A Molecular Dynamics Study
Source: PLoS One. 2014 May 9;9(5):e96660. doi: 10.1371/journal.pone.0096660 (PMC4015988; doi:10.1371/journal.pone.0096660)
Supplement: Figure S8 — a Average number of backbone-backbone H-bonds between pairs of peptides in stable systems. Name of the simulation is within each panel. b Average number of side chain-side chain H-bonds between pair of peptides in stable systems with neutral termini. Name of the simulation is within each panel. c Average number of backbone-backbone H-bonds between pairs of peptides in the extended simulations (top and middle panel) and re-initiated simulations (bottom panel). Name of the simulation is within each panel. d Average number of side chain-side chain H-bonds between pairs of peptides in between pairs of peptides in the extended simulations (top and middle panel) and re-initiated simulations (bottom panel). Name of the simulation is within each panel. (PDF) [file pone.0096660.s008.pdf]

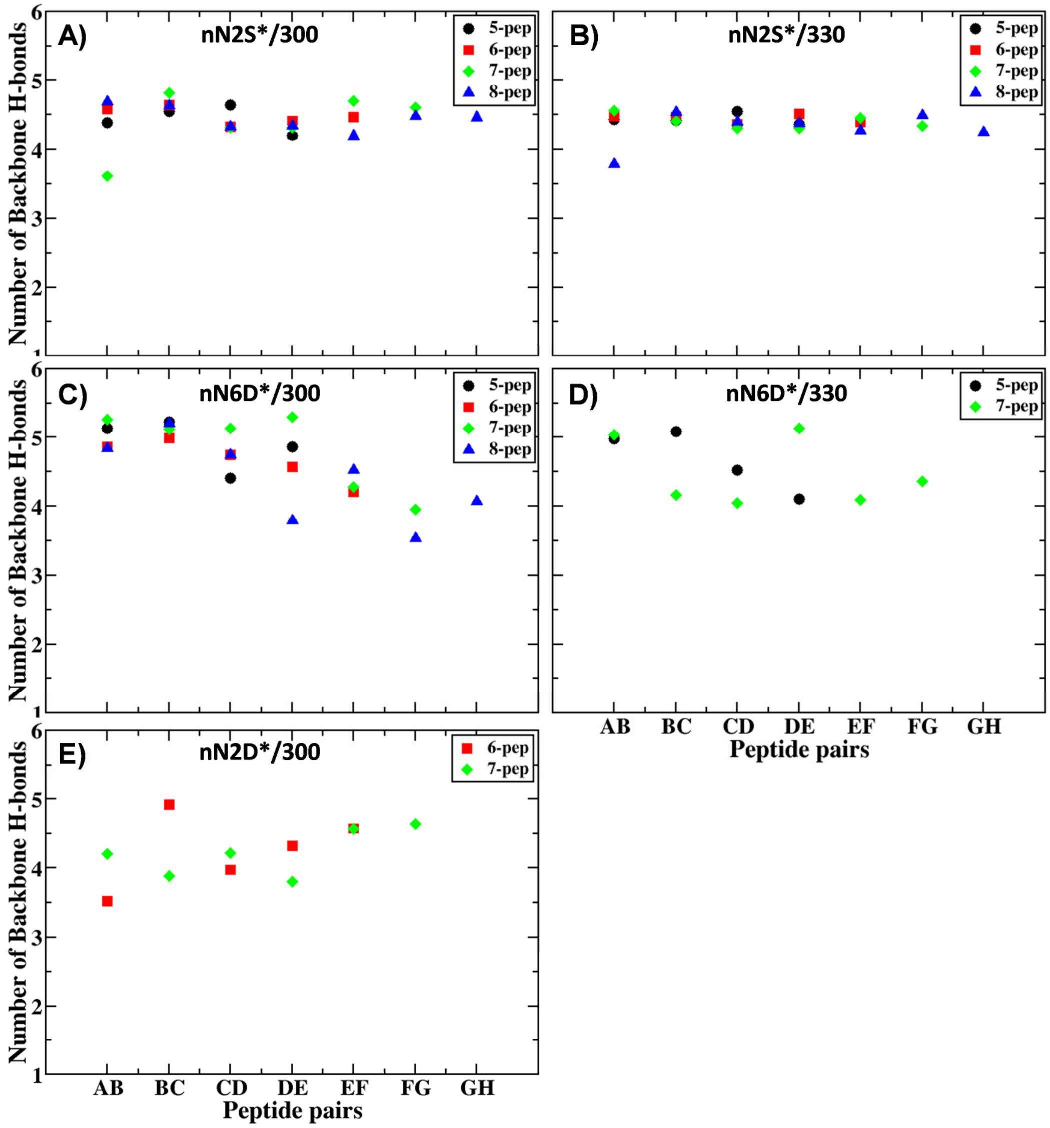

Figure S8a Average number of backbone-backbone H-bonds between pairs of peptides in stable systems. Name of the simulation is within each panel.

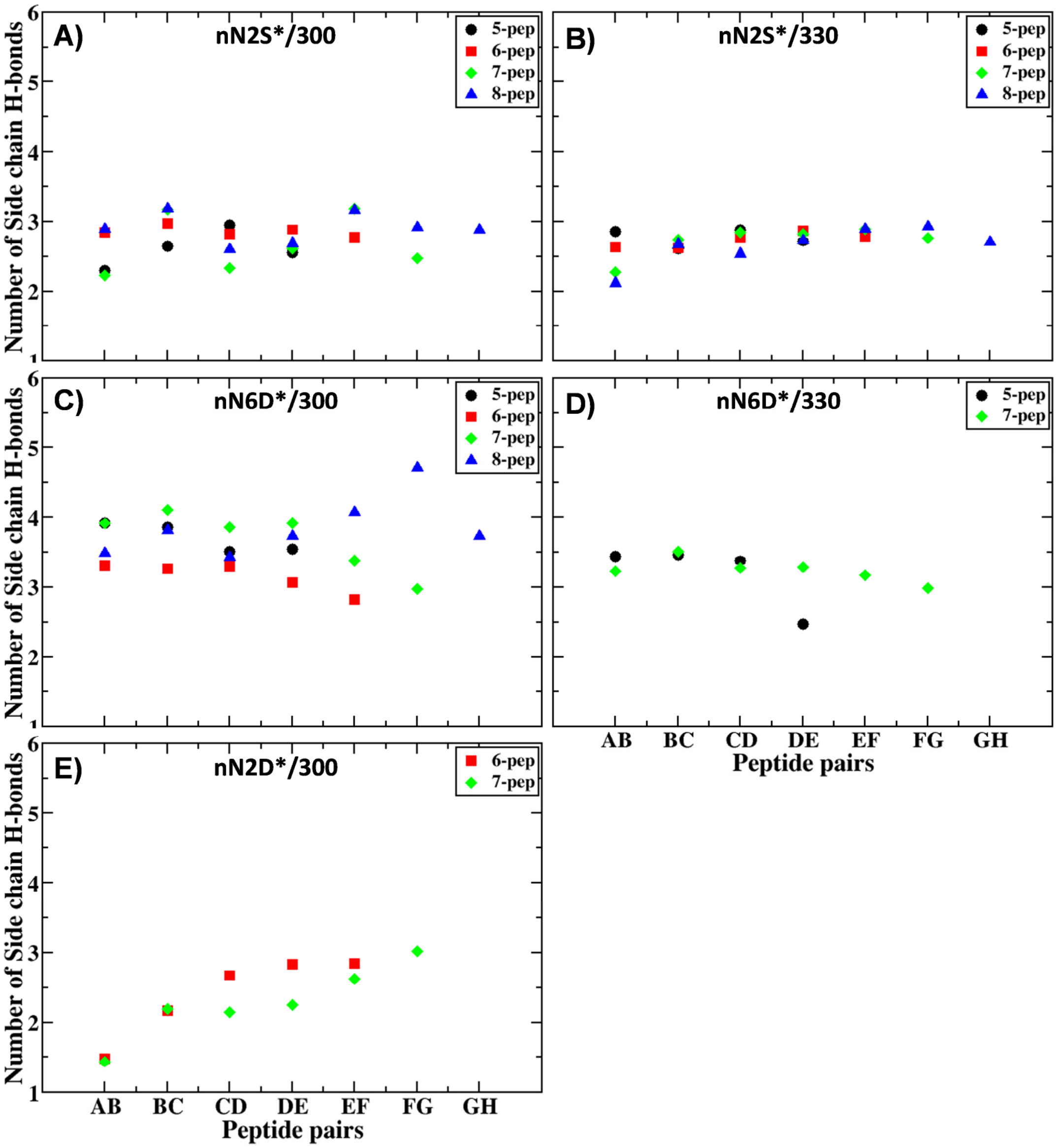

Figure S8b Average number of side chain-side chain H-bonds between pair of peptides in stable systems with neutral termini. Name of the simulation is within each panel.

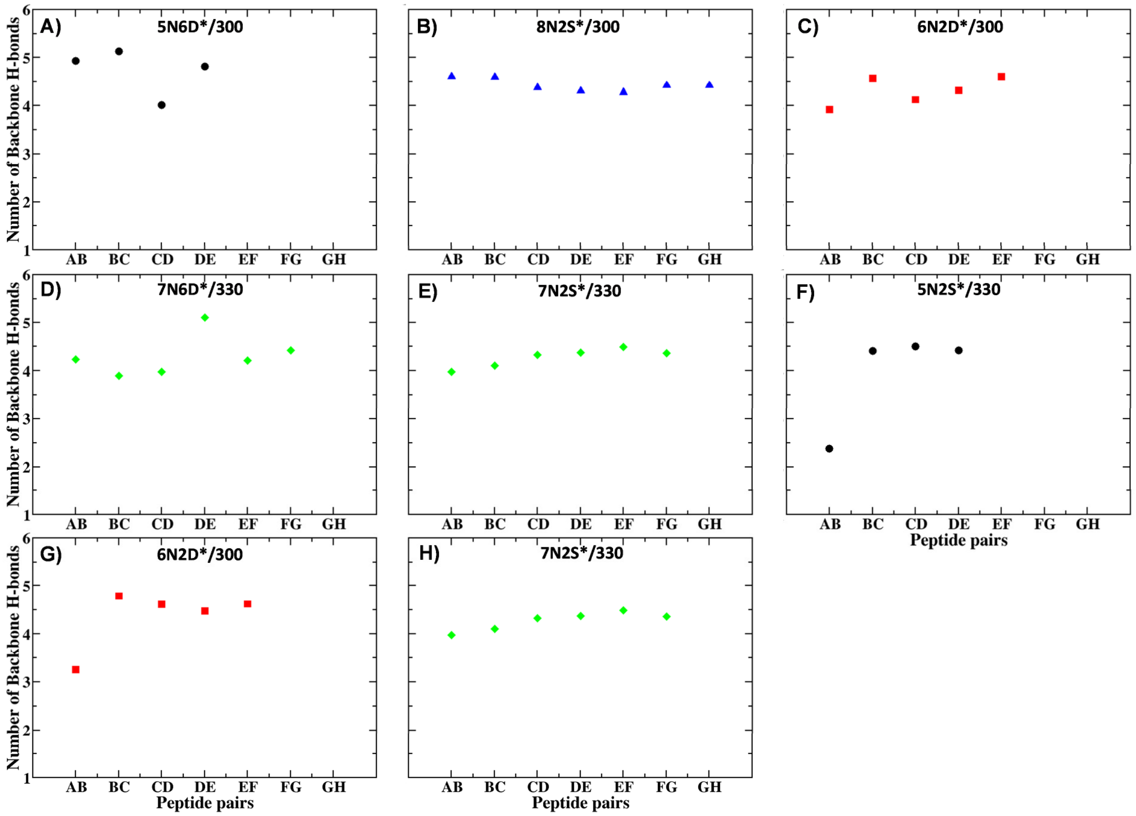

Figure S8c Average number of backbone-backbone H-bonds between pairs of peptides in the extended simulations (top and middle panel) and re-initiated simulations (bottom panel). Name of the simulation is within each panel.

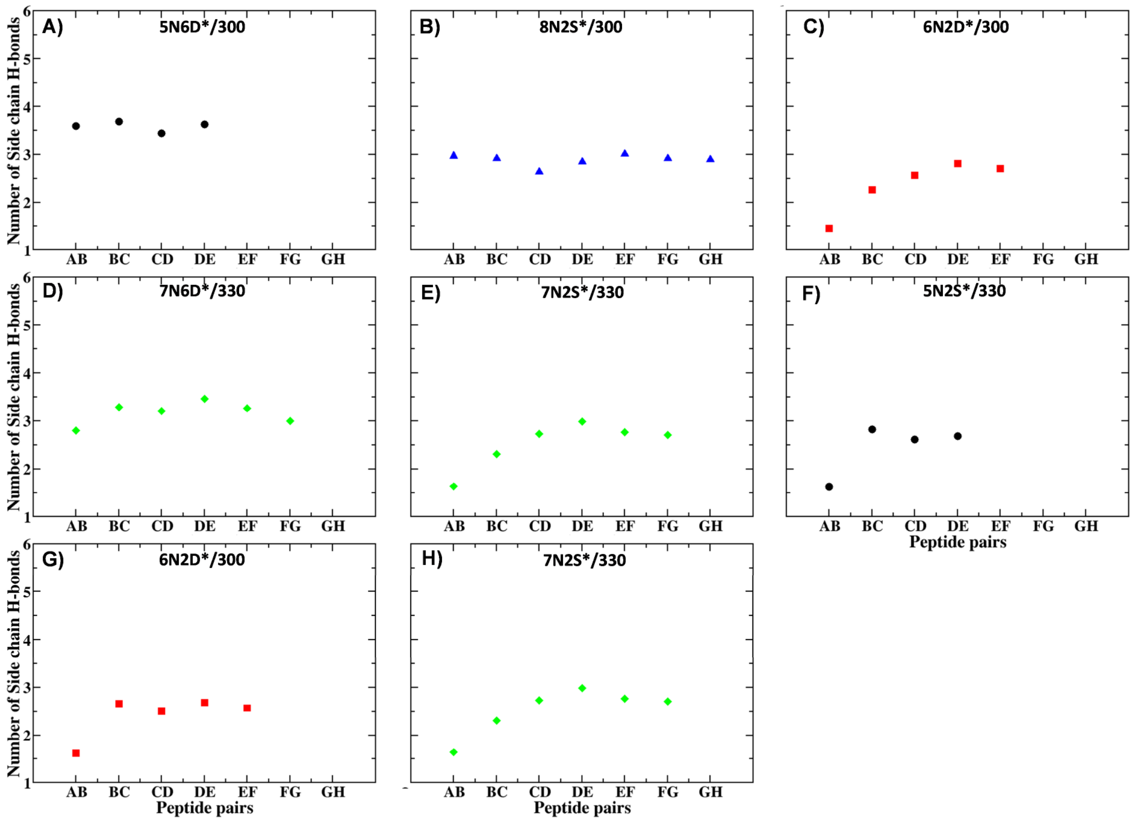

Figure S8d Average number of side chain-side chain H-bonds between pairs of peptides in between pairs of peptides in the extended simulations (top and middle panel) and re-initiated simulations (bottom panel). Name of the simulation is within each panel.
