# Supplementary material for: Interplay of Sequence, Topology and Termini Charge in Determining the Stability of the Aggregates of GNNQQNY Mutants: A Molecular Dynamics Study
Source: PLoS One. 2014 May 9;9(5):e96660. doi: 10.1371/journal.pone.0096660 (PMC4015988; doi:10.1371/journal.pone.0096660)
Supplement: Figure S9 — Comparison of the total number of backbone and side chain H-bonds in mutant aggregates with those in WT aggregates (data from ref 57). Data averaged over all the peptides and entire trajectory. Data compared for only the stable systems. (PDF) [file pone.0096660.s009.pdf]

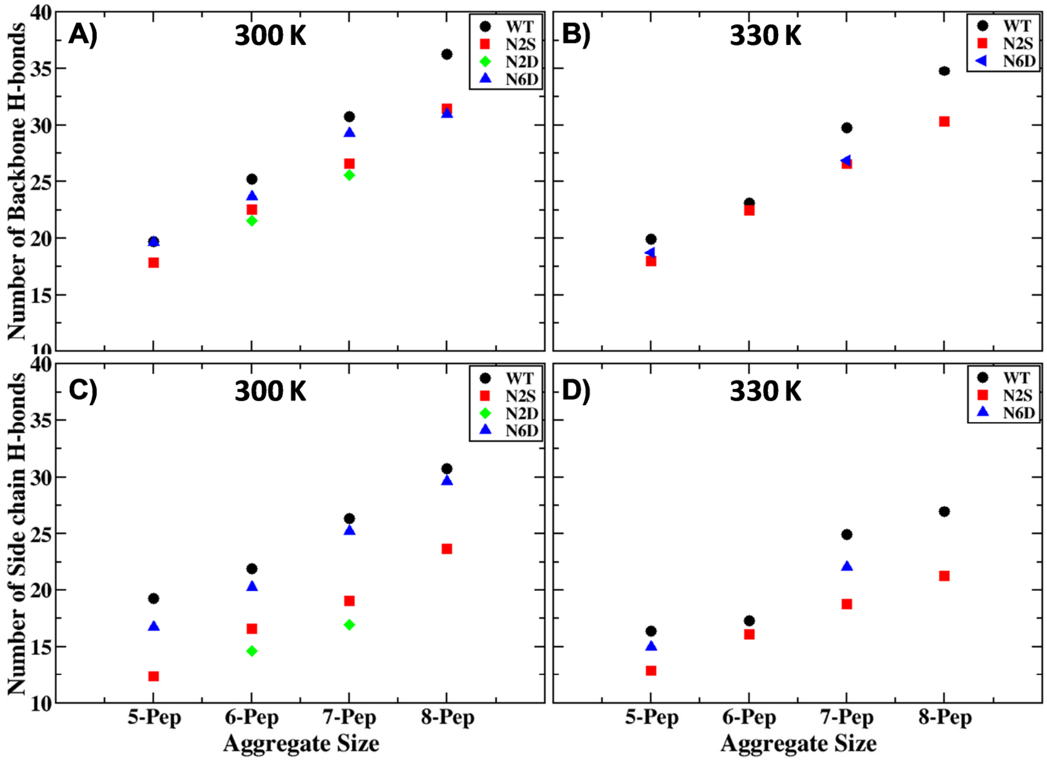

Figure S9 Comparison of the total number of backbone and side chain H-bonds in mutant aggregates with those in WT aggregates (data from ref 57). Data averaged over all the peptides and entire trajectory. Data compared for only the stable systems.
