# Supplementary material for: Interplay of Sequence, Topology and Termini Charge in Determining the Stability of the Aggregates of GNNQQNY Mutants: A Molecular Dynamics Study
Source: PLoS One. 2014 May 9;9(5):e96660. doi: 10.1371/journal.pone.0096660 (PMC4015988; doi:10.1371/journal.pone.0096660)
Supplement: Figure S10 — a Average number of backbone-backbone H-bonds formed by different residues of the peptide in N2S* systems. Name of the simulation is within each panel. Data for edge peptides, which have only one partner, have not been shown. b Average number of side chain-side chain H-bonds formed by different residues of the peptide in N2S* systems. Name of the simulation is within each panel. Data for edge peptides, which have only one partner, have not been shown. c Average number of backbone-backbone and side chain-side chain H-bonds formed by different residues of the peptide in stable N2D* systems. Name of the simulation is within each panel. Data for edge peptides, which have only one partner to interact with, have not been shown. d Average number of backbone-backbone H-bonds formed by different residues of the peptide in the stable N6D* systems. Name of the simulation is within each panel. Data for edge peptides, which have only one partner to interact with, have not been shown. e Average number of side chain-side chain H-bonds formed by different residues of the peptide in the stable N6D* systems. Name of the simulation is within each panel. Data for edge peptides, which have only one partner to interact with, have not been shown. f Average number of backbone-backbone H-bonds formed by different residues of the peptide in between pairs of peptides in the extended simulations (top and middle panel) and re-initiated simulations (bottom panel). Name of the simulation is within each panel. Data for edge peptides, which have only one partner, have not been shown. g Average number of side chain-side chain H-bonds formed by different residues of the peptide in in between pairs of peptides in the extended simulations (top and middle panel) and re-initiated simulations (bottom panel). Name of the simulation is within each panel. Data for edge peptides, which have only one partner, have not been shown. (PDF) [file pone.0096660.s010.pdf]

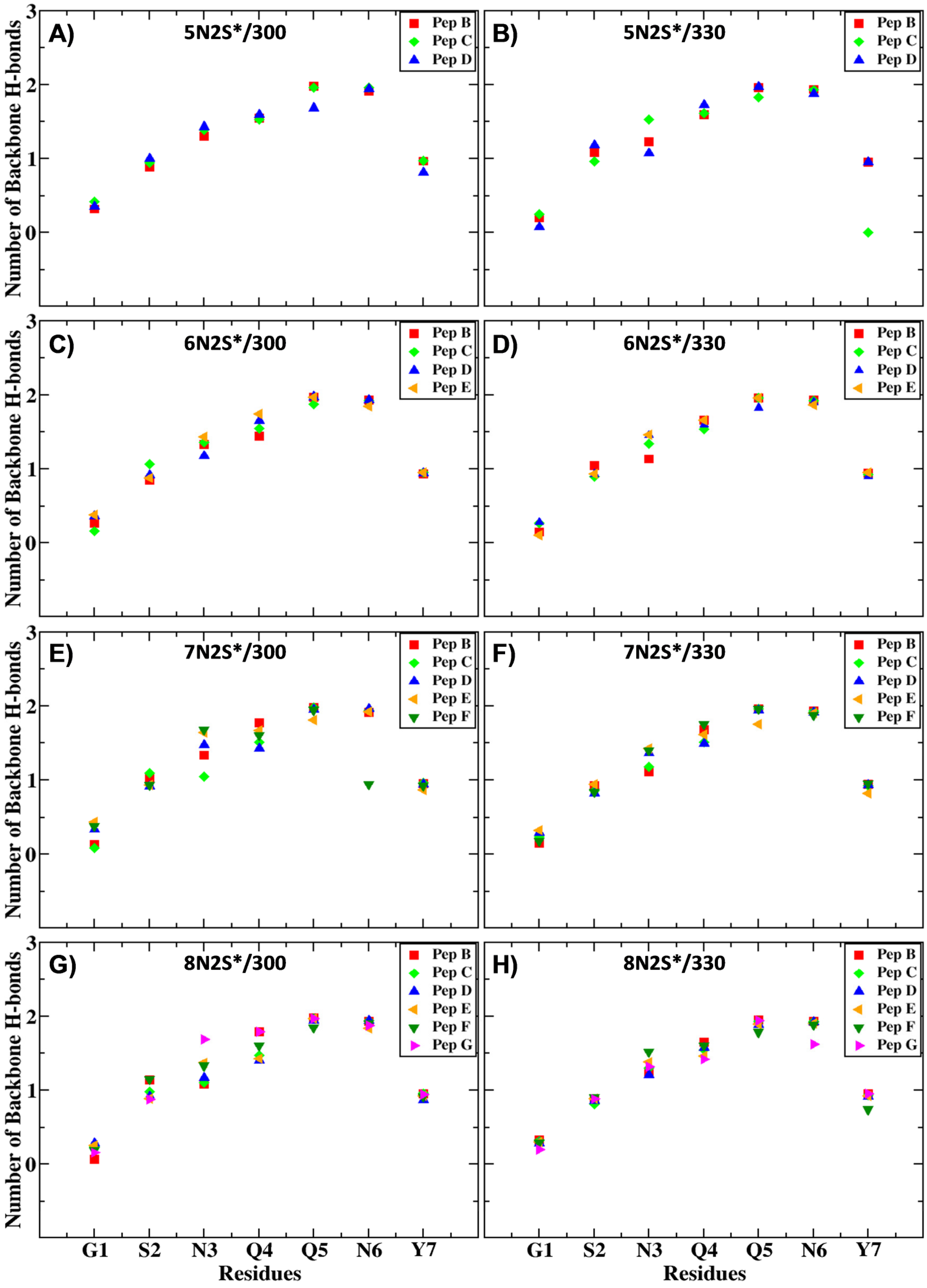

Figure S10a Average number of backbone-backbone H-bonds formed by different residues of the peptide in N2S* systems. Name of the simulation is within each panel. Data for edge peptides, which have only one partner, have not been shown.

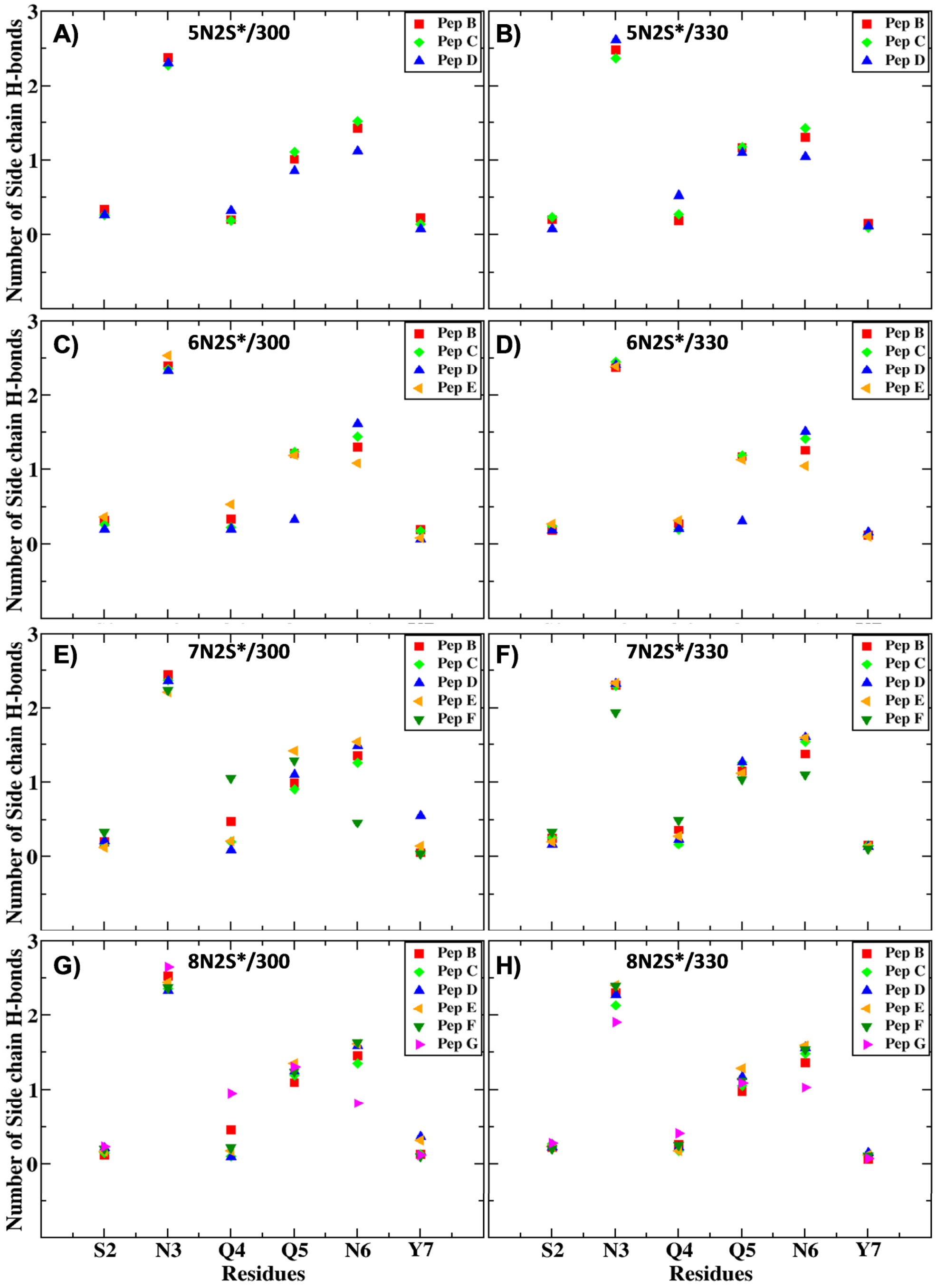

Figure S10b Average number of side chain-side chain H-bonds formed by different residues of the peptide in N2S* systems. Name of the simulation is within each panel. Data for edge peptides, which have only one partner, have not been shown.

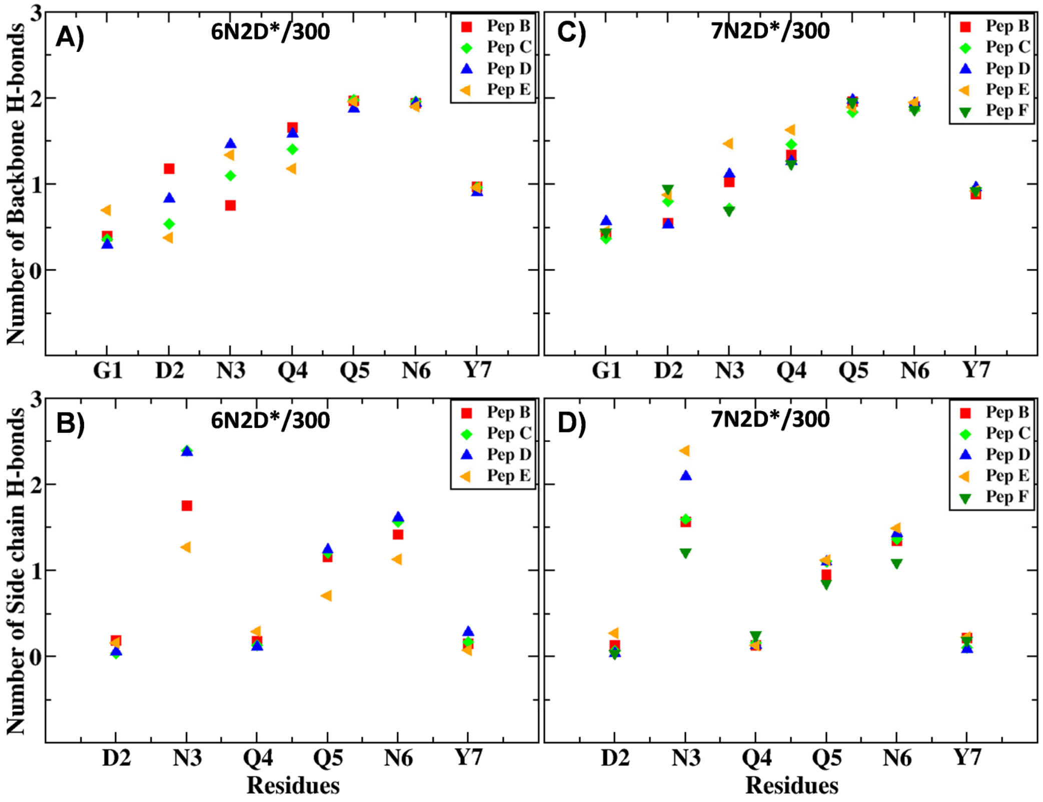

Figure S10c Average number of backbone-backbone and side chain-side chain H-bonds formed by different residues of the peptide in stable N2D* systems. Name of the simulation is within each panel. Data for edge peptides, which have only one partner to interact with, have not been shown.

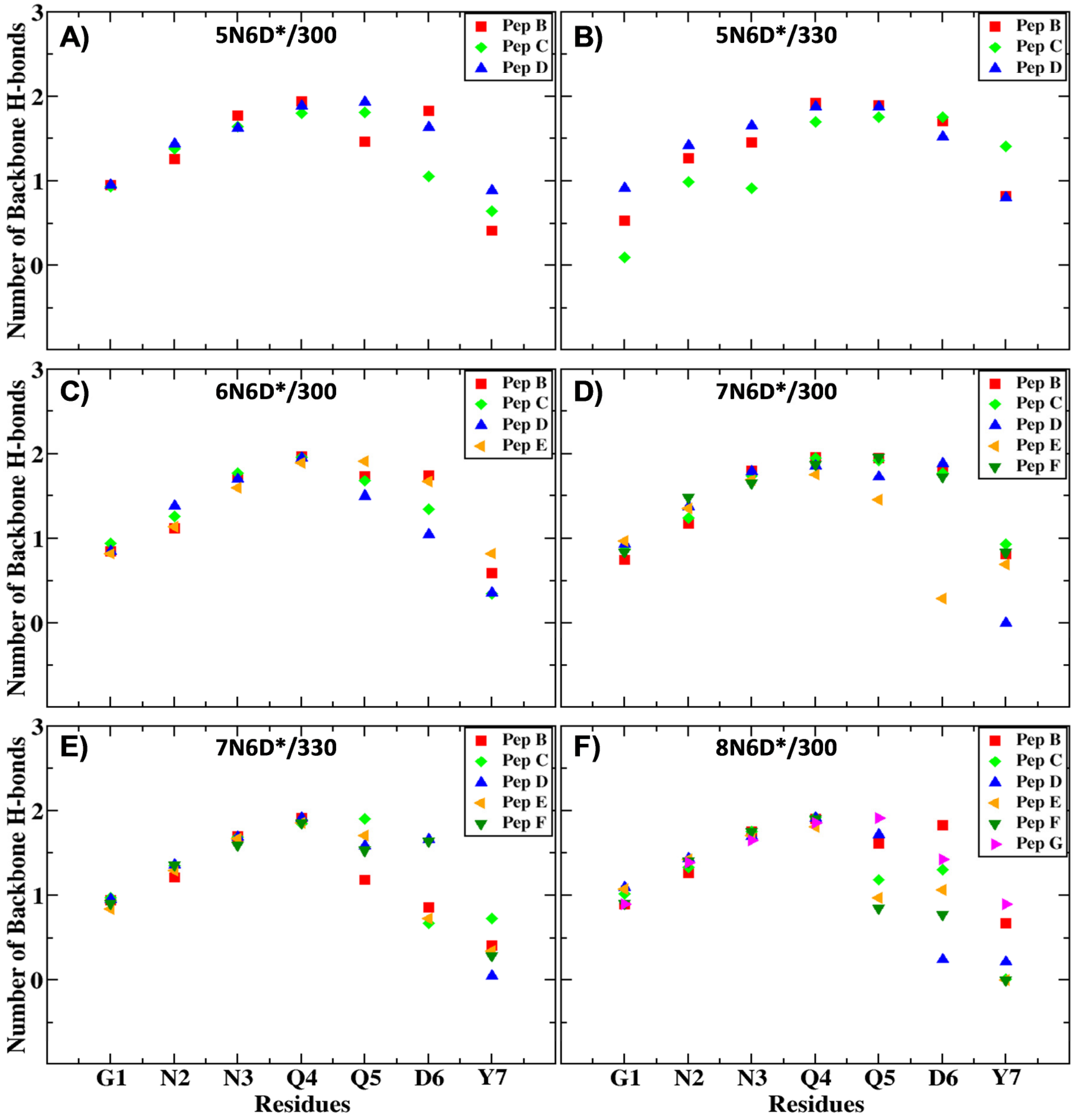

Figure S10d Average number of backbone-backbone H-bonds formed by different residues of the peptide in the stable N6D* systems. Name of the simulation is within each panel. Data for edge peptides, which have only one partner to interact with, have not been shown.

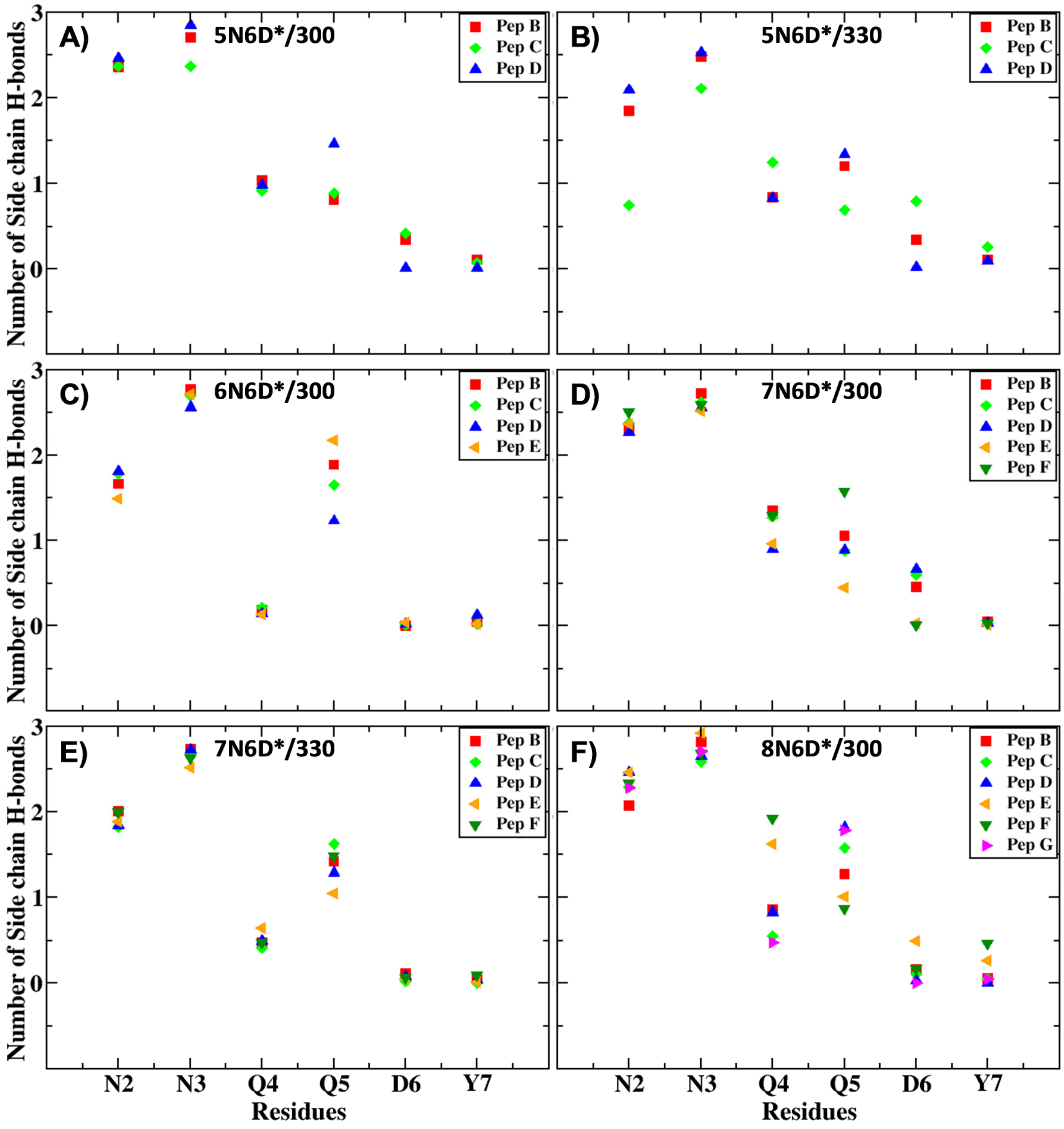

Figure S10e Average number of side chain-side chain H-bonds formed by different residues of the peptide in the stable N6D* systems. Name of the simulation is within each panel. Data for edge peptides, which have only one partner to interact with, have not been shown.

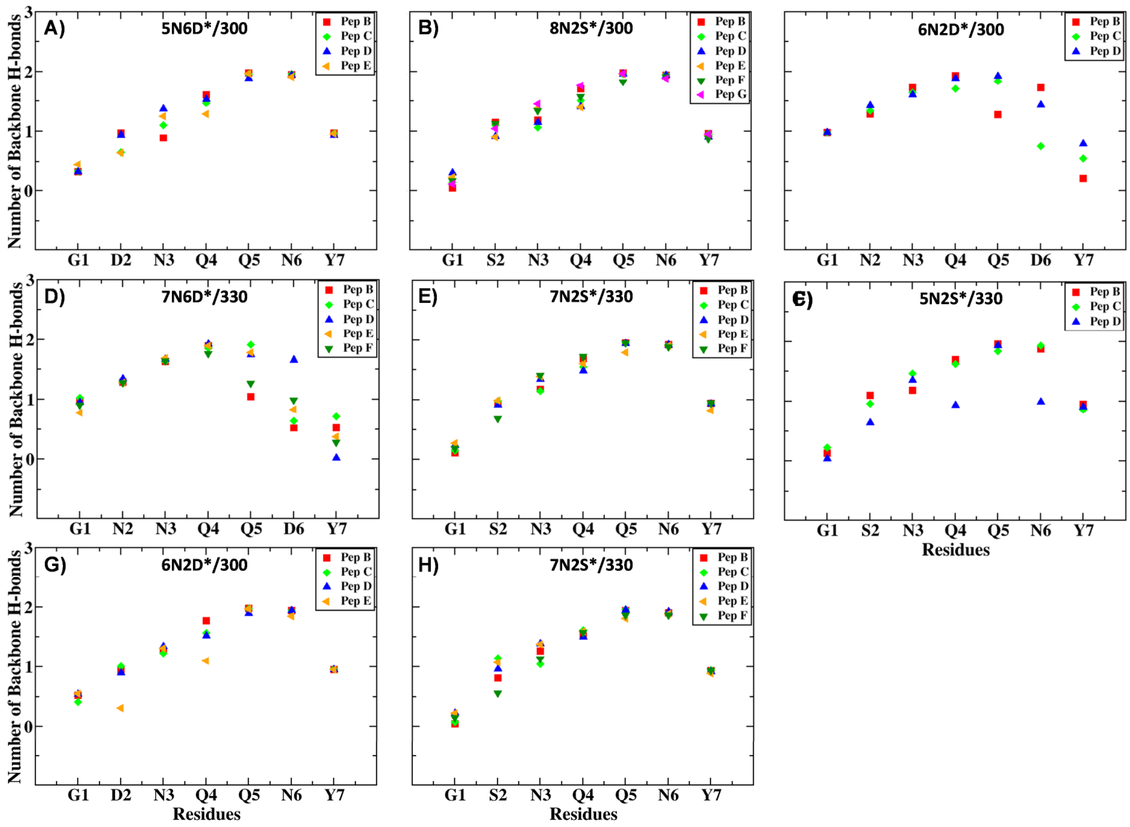

Figure S10f Average number of backbone-backbone H-bonds formed by different residues of the peptide in between pairs of peptides in the extended simulations (top and middle panel) and re-initiated simulations (bottom panel). Name of the simulation is within each panel. Data for edge peptides, which have only one partner, have not been shown.

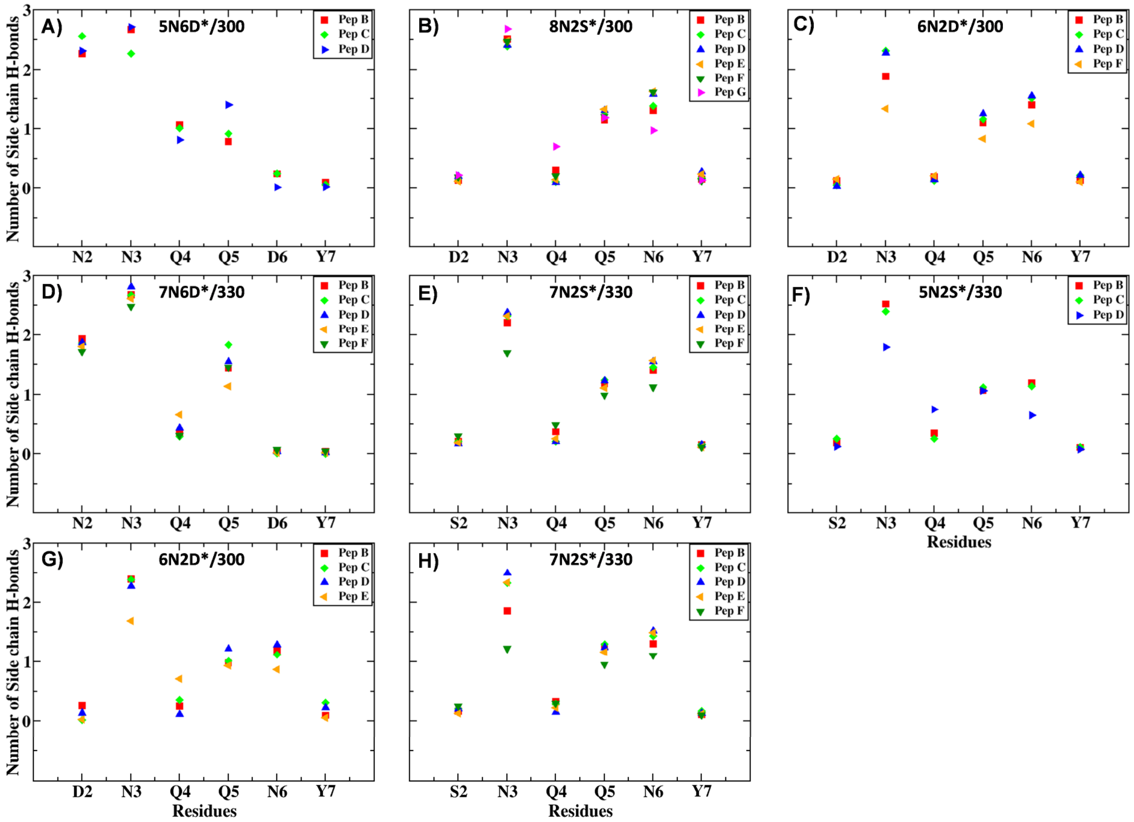

Figure S10g Average number of side chain-side chain H-bonds formed by different residues of the peptide in in between pairs of peptides in the extended simulations (top and middle panel) and re-initiated simulations (bottom panel). Name of the simulation is within each panel. Data for edge peptides, which have only one partner, have not been shown.
