# Supplementary material for: Interplay of Sequence, Topology and Termini Charge in Determining the Stability of the Aggregates of GNNQQNY Mutants: A Molecular Dynamics Study
Source: PLoS One. 2014 May 9;9(5):e96660. doi: 10.1371/journal.pone.0096660 (PMC4015988; doi:10.1371/journal.pone.0096660)
Supplement: Table S1 — Summary of events observed in unstable systems. (PDF) [file pone.0096660.s011.pdf]

Table S1 Summary of events observed in unstable systems

EVENTS	SYSTEMS
Formation of an amorphous aggregate consisting of a central sheetsurrounded by peptides	5N2S/300,7N2S/330, 5N2D/330, 7N2D/330, 5N6D/300, 8N6D/300, 8N6D/330
Breakage	
Smaller aggregates move away from one other	6N2S/330, 6N6D/300
Smaller aggregates form a rather tight cluster	8N2S/330
Smaller aggregates form a lose aggregate	6N6D/330
Disorder followed by dissociation of edge peptides	
Dissociated peptide moves away from the aggregate	5N2S/330 (peptide A), 7N2S/300 (G), 7N2S/330 (G),6N2D/300(A), 6N2D/330 (A), 7N2D/300 (A),8N2D/300 (H), 7N6D/300 (A), 8N6D/300 (F), 6N6D*/330 (F), 7N2D*/330(G), 8N6D*/330 (H), 5N2S*/330 (A)
Dissociated peptide associates loosely with the neighbouring peptides	6N2S/300 (F), 8N2S/300 (H), 6N2D/330 (F), 8N6D*/330 (H), 6N2D*/330 (A and B with C)
Dissociated peptide spreads on top of the aggregate	5N2S/330 (E), (6N2S/300 (F), 7N2S/300 (G), 7N2D/300 (A), 8N2D/300 (A and H), 8N6D*/330 (H), 5N2S*/330 (A)
Dissociated peptide re-associates with the aggregate from the opposite end	5N2S/330 (A), 7N2D/300 (A)
Dissociated peptide re-associates with the aggregate in an amorphous manner	7N2S/330 (G), 8N6D*/330 (H)
Edge peptide is attached to its neighbor through only one or two residues	5N2D*/300, 5N2D*/330, 6N6D*/330
Parallel to anti-parallel transition of a peptide or a 2-mer	6N2S/300, 6N2S/330, 7N2S/300, 8N2S/300, 5N2D/300, 5N2D/330, 7N2D/300, 8N2D/300, 6N6D/300, 7N6D/300, 5N6D*/330, 8N2D*/330
Formation of a double layer-like arrangement	7N2D/300, 8N2D/300, 8N2D*/300
