# Supplementary material for: Interplay of Sequence, Topology and Termini Charge in Determining the Stability of the Aggregates of GNNQQNY Mutants: A Molecular Dynamics Study
Source: PLoS One. 2014 May 9;9(5):e96660. doi: 10.1371/journal.pone.0096660 (PMC4015988; doi:10.1371/journal.pone.0096660)
Supplement: Table S2 — Sizes of the smaller ordered aggregates that form in unstable systems. (PDF) [file pone.0096660.s012.pdf]

Table S2 Sizes of the smaller ordered aggregates that form in unstable systems

SYSTEM	SIZE	SYSTEM	SIZE	SYSTEM	SIZE
5N2D/300	2-mer	5N6D/300	2-mer	5N2S/300	2-mer
5N2D/330	2-mer	5N6D/330	2-mer, 3-mer	5N2S/330	2-mer, 3-mer
6N2D/300	4-mer, 3-mer	6N6D/300	Two 2-mer	6N2S/300	2-mer, 3-mer, two 2-mer
6N2D/330	4-mer, 2-mer	6N6D/330	2-mer, 3-mer	6N2S/330	2-mer, 3-mer, 4-mer
7N2D/300	4-mer, 5-mer	7N6D/300	2-mer	7N2S/300	2-mer, 3-mer, two 2-mer
7N2D/330	2-mer	7N6D/330	2-mer	7N2S/330	2-mer
8N2D/300	6-mer	8N6D/300	2-mer	8N2S/300	2-mer, 3-mer, 4-mer
8N2D/330	6-mer, 5-mer	8N6D/330	2-mer	8N2S/330	3-mer, 2-mer
5N2D*/300	4-mer	5N6D*/330	5-mer	5N2S*/330	4-mer
5N2D*/330	4-mer	6N6D*/330	2-mer and 3-mer		
6N2D*/330	4-mer, 3-mer	8N6D*/330	7-mer		
7N2D*/330	6-mer				
8N2D*/300	6-mer				
8N2D*/330	7-mer, 8-mer				
