# Supplementary material for: Interplay of Sequence, Topology and Termini Charge in Determining the Stability of the Aggregates of GNNQQNY Mutants: A Molecular Dynamics Study
Source: PLoS One. 2014 May 9;9(5):e96660. doi: 10.1371/journal.pone.0096660 (PMC4015988; doi:10.1371/journal.pone.0096660)
Supplement: Table S3 — Comparison of the structural characteristics of smaller aggregates formed by unstable systems of mutant peptides with the microcrystal structure of GNNQQNY. (PDF) [file pone.0096660.s013.pdf]

Table S3 Comparison of the structural characteristics of smaller aggregates formed by unstable systems of mutant peptides with the microcrystal structure of GNNQQNY[¶]

Smaller aggregates	Microcrystal
Peptide-peptide distance [ŧ]: 0.47 nm	~0.48 nm
Twist exists both within and across peptides	Planar sheet
Some strands are anti-parallel	All strands are parallel
Only non-terminal residues in sheet conformation[§]	All residue in sheet conformation
S2, D2, rarely in sheet conformation	
Tyr-Tyr stacking absent	Tyr-Tyr stacking present
Only some backbone NH and CO form H-bonds	All backbone NH and CO groups form H-bonds
Side-chain H-bonds are fewer than those in the microcrystal structure	

[ŧ]Only those residues that are in sheet are considered for distance calculation.

[§]3 to 5 residues in neutral termini peptides; 2 to 4 residues in charged termini peptides are in sheet.

[¶]Data from Ref. 26.
