# Supplementary material for: Interplay of Sequence, Topology and Termini Charge in Determining the Stability of the Aggregates of GNNQQNY Mutants: A Molecular Dynamics Study
Source: PLoS One. 2014 May 9;9(5):e96660. doi: 10.1371/journal.pone.0096660 (PMC4015988; doi:10.1371/journal.pone.0096660)
Supplement: Table S4 — Comparison of the stable systems with the Wild Type systems and microcrystal structure of GNNQQNY. (PDF) [file pone.0096660.s014.pdf]

Table S4 Comparison of the stable systems with the Wild Type systems and microcrystal structure of GNNQQNY€

	N2S	N2D	N6D	WT£	Microcrystal€
Twist¥	15 to 25°	15 to 30°	10 to 30°	15 to 25°	Planar
End Fraying COM§or Cα-Cα¥	G1 = 0.6 to 0.8 nm<br>S2 to Y7 ≤ 0.5 nm	G1 to N3 = 0.5 to 0.9 nm<br>Q4 to Y7 ≤ 0.5 nm	G1 = 0.5 to 0.6 nm<br>N2 to Q4 ≤ 0.5 nm<br>D6 to Y7 = 0.5 to 0.8 nm	COM_d~0.47 nm	Exactly in-register strands,<br>d§~ 0.48 nm
Tyr distance	Mostly around 0.5 nm	Mostly around 0.5 nm	Mostly > 0.5 nm (High Fluctuation)	Mostly around 0.5 nm	Stacking observed
Sheet Conformations	G1, Y7 = never<br>S2 = less frequently<br>N3 to N6 =almost always	G1, Y7 = never<br>D2 = less frequently<br>N3 to N6 = almost always	G1, Y7 = never<br>D6 = frequently<br>N2 to Q5 = almost always	G1, Y7 = never<br>Rest = almost always	All residues in sheet
H-bonds¥	Backbone = 4 to 5<br>Side chain = 2 to 3	Backbone = 3 to 4<br>Side chain < 3	Backbone = 3 to 6<br>Side chain = 2 to 5	Backbone = 4 to 6<br>Side chain = 3 to 6	Backbone = 5<br>Side chain = 5

£The data for wild type systems are taken from Srivastava and Balaji [ref. 57] and are mentioned here for comparison.
€The values are taken from the microcrystal structure of GNNQQNY reported by Nelson *et. al.,*[ref. 26] and are mentioned here for comparison.
¥ Average values is given for these parameters and the range is spanning from 5 to 8 peptide systems for the mutant and WT systems.
§ For wild type systems centre of mass distance (COM_d) between pair of peptides is given and for microcrystal inter-strand distance is given.
